# The Smc5/6 complex regulates the yeast Mph1 helicase at RNA-DNA hybrid-mediated DNA damage

**DOI:** 10.1371/journal.pgen.1007136

**Published:** 2017-12-27

**Authors:** Juan Lafuente-Barquero, Sarah Luke-Glaser, Marco Graf, Sonia Silva, Belén Gómez-González, Arianna Lockhart, Michael Lisby, Andrés Aguilera, Brian Luke

**Affiliations:** 1 Andalusian Center for Molecular Biology and Regenerative Medicine-CABIMER, Universidad de Sevilla-CSIC-Universidad Pablo de Olavide, Avda. Americo Vespucio 24, Seville, Spain; 2 Institute of Molecular Biology (IMB), Mainz, Germany; 3 Zentrum für Molekulare Biologie der Universität Heidelberg (ZMBH), DKFZ-ZMBH Alliance, Heidelberg, Germany; 4 Department of Biology, University of Copenhagen, Ole Maaloeesvej 5, Copenhagen N, Denmark; 5 Institute of Neurobiology and Developmental Biology, JGU Mainz, Mainz, Germany; Georgia Institute of Technology, UNITED STATES

## Abstract

RNA-DNA hybrids are naturally occurring obstacles that must be overcome by the DNA replication machinery. In the absence of RNase H enzymes, RNA-DNA hybrids accumulate, resulting in replication stress, DNA damage and compromised genomic integrity. We demonstrate that Mph1, the yeast homolog of Fanconi anemia protein M (FANCM), is required for cell viability in the absence of RNase H enzymes. The integrity of the Mph1 helicase domain is crucial to prevent the accumulation of RNA-DNA hybrids and RNA-DNA hybrid-dependent DNA damage, as determined by Rad52 foci. Mph1 forms foci when RNA-DNA hybrids accumulate, e.g. in RNase H or THO-complex mutants and at short telomeres. Mph1, however is a double-edged sword, whose action at hybrids must be regulated by the Smc5/6 complex. This is underlined by the observation that simultaneous inactivation of RNase H2 and Smc5/6 results in Mph1-dependent synthetic lethality, which is likely due to an accumulation of toxic recombination intermediates. The data presented here support a model, where Mph1’s helicase activity plays a crucial role in responding to persistent RNA-DNA hybrids.

## Author summary

DNA damage can either occur exogenously through DNA damaging agents such as UV light and exposure to chemotherapeutics, or endogenously via metabolic, cellular processes. The RNA product of transcription, for example, can engage in the formation of RNA-DNA hybrids. Such RNA-DNA hybrids can impede replication fork progression and cause genomic instability, a hallmark of cancer. The misregulation of RNA-DNA hybrids has also been implicated in several neurological disorders. Recently, it has become evident that RNA-DNA hybrids may also have beneficial roles and therefore, these structures have to be tightly controlled. We found that Mph1 (mutator phenotype 1), the budding yeast homolog of Fanconi Anemia protein M, counteracts the accumulation of RNA-DNA hybrids. The inactivation of *MPH1* results in a severe growth defect when combined with mutations in the well-characterized RNase H enzymes, that degrade the RNA moiety of an RNA-DNA hybrid. Based on the data presented here, we propose a model, where Mph1 itself has to be kept in check by the SMC (structural maintenance of chromosome) 5/6 complex at replication forks stalled by RNA-DNA hybrids. Mph1 acts as a double-edged sword, as both its deletion and the inability to control its helicase activity cause DNA damage and growth arrest when RNA-DNA hybrids accumulate.

## Introduction

RNA-DNA hybrids can form during transcription when the nascent RNA base pairs with the template strand of the DNA. This results in a 3-stranded structure, referred to as an R-loop [1, 2]. R-loops lead to DNA replication stress and hence increased rates of DNA breakage, unscheduled recombination, and genome rearrangements [3, 4]. It is unclear exactly how R-loops cause replication stress, but it may be linked to their ability to promote local chromatin compaction [5]. R-loops can be removed by the RNase H enzymes (RNase H1 and RNase H2), which can degrade the RNA moiety of an RNA-DNA hybrid molecule. In yeast, RNase H1 is encoded by the *RNH1* gene, while the RNase H2 enzyme is a trimeric complex made up of the gene products of *RNH201* (the catalytic subunit), *RNH202*, and *RNH203* [6, 7]. Mutations in all three subunits of RNase H2 have been linked to the neurological auto-immune disorder, Aicardi-Goutières syndrome (AGS) [8].

In addition to RNase H-mediated hybrid degradation, the THO complex counteracts the accumulation of RNA-DNA hybrids. The THO complex is a transcription-coupled RNA processing complex that limits the formation of RNA-DNA hybrids in a co-transcriptional manner [9] by “capturing” the nascent RNA and ensuring that it gets efficiently exported. Furthermore, multiple helicases have been implicated in RNA-DNA hybrid removal via displacement of the RNA strand, including yeast Pif1 [10] and Sen1 [11], human SETX (the equivalent of yeast Sen1) [12], AQR [13], DDX19 [14], DDX23 [15], DDX1 [16] and DDX21 [17]. When R-loop removal factors are inactivated, many DNA repair genes (namely those involved in homologous recombination) become essential, underlining the propensity of R-loops to promote DNA damage as a result of replication stress [1, 2]. RNA-DNA hybrids exist at telomeres in yeast and human cells and promote homology-directed repair [18–20] demonstrating their involvement in telomere maintenance. As telomeres shorten, RNA-DNA hybrids accumulate together with increased levels of the long non-coding RNA, telomeric repeat-containing RNA (TERRA)[21–25], suggesting that telomeric RNA-DNA hybrids are made up of TERRA.

The recent observations that the Fanconi Anemia repair pathway, which is involved in the repair of DNA inter-strand crosslinks and obstacles that impede replication fork progression, is crucial to dissolve R-loops that may block replication forks in human cells [26, 27] suggest a relevant role of this DNA repair pathway in R-loop prevention and removal. Human FANCM has the ability to branch migrate replication structures, resolve RNA-DNA hybrids *in vitro* as well as to prevent RNA-DNA hybrid accumulation *in viv*o in human and chicken cells [27]. Although budding yeast cells lack a canonical Fanconi Anemia pathway, yeast Mph1 helicase stands out as a homolog of the FANCM protein [28]. *In vitro*, Mph1 is able to dissociate D-loops and promote replication fork reversal, similarly to FANCM [29]. Both the deletion and overexpression of yeast Mph1 are associated with increased replication stress and genome instability [30, 31], respectively indicating that its activity is important when facing replication stress, but must be tightly regulated in order to prevent toxicity. Interestingly, the loss of Mph1 has been reported to lead to a synthetic growth defect in the absence of *RNH203* [32], pointing to a possible role for Mph1 in R-loop regulation.

Several studies have reported that the Smc5/6 complex is a negative regulator of Mph1 [33–35]. Two SMC subunits (Smc5 and Smc6) and 6 non-SMC elements form this highly conserved complex, which structurally resembles the cohesin, condensin and the MRN complexes [36]. The deletion of *MPH1* is able to rescue the temperature sensitive growth defect of *smc5/6* mutant alleles as well as the accumulation of X-shaped recombination intermediates after treatment with DNA damaging agents [33–35]. Interestingly, the inactivation of the Smc5/6 complex [37, 38], as well as overexpression of Mph1 [39], have been demonstrated to drive yeast into premature replicative senescence.

Given the observations outlined above, we set out to investigate the role of Mph1 and its established negative regulator, Smc5/6 at RNA-DNA hybrids *in vivo* in the budding yeast *Saccharomyces cerevisiae*. In this study, we show that Mph1 forms nuclear foci when RNA-DNA hybrids accumulate, i.e. in *rnh1 rnh201* mutants and at short telomeres. We demonstrate that Mph1, and in particular its helicase function, suppresses the accumulation of RNA-DNA hybrids, at RNAPII-transcribed genes as well as at telomeres, and that in the absence of Mph1, cells accumulate recombinogenic DNA damage in an RNA-DNA hybrid-dependent manner. Accordingly, Mph1 becomes essential when RNA-DNA hybrid removal is strongly impaired, as seen by the severe growth defect of the *rnh1 rnh201 mph1* triple mutants. However, when the functionality of the Smc5/6 complex is compromised, Mph1’s helicase activity becomes deleterious in situations when RNA-DNA hybrids accumulate. We propose that Mph1 plays an important role in RNA-DNA hybrid metabolism and that its activity has to be tightly controlled by the Smc5/6 complex.

## Results

### Mph1 prevents the accumulation of hybrid-induced Rad52 foci

**Fig 1:**
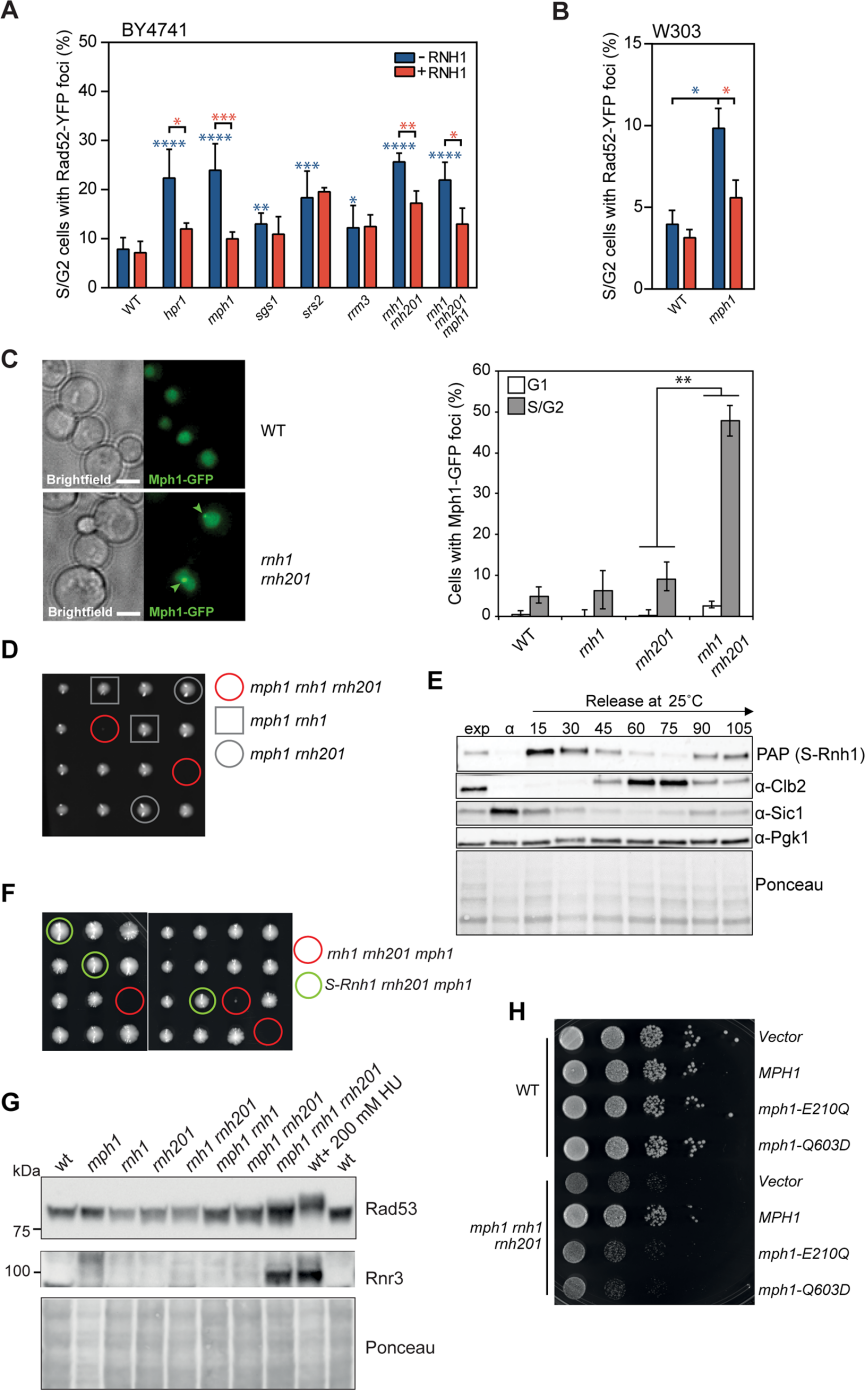
Mph1 helicase domain prevents the accumulation of Rad52 foci, and is required in the absence of RNase H1 and RNase H2. **A.** Quantification of spontaneous Rad52-YFP foci formation in WT and candidate mutant helicase strains as well as the double mutant *rnh1 rnh201* (ySLG428) and triple mutant *rnh1 rnh201 mph1* (ySLG611) (BY4741 background). Cells were transformed with pWJ1314, that carries the Rad52-YFP fusion, and the empty plasmid pCM189 (-RNH) or pCM189::RNH1 (+RNH). Average and SD of three to six independent experiments are shown. **B.** Rad52-GFP foci accumulation in WT (W303.1A) and *mph1* (WMPH.2B) mutant in the W303 background, with and without over-expression of RNH. * P<0.05; ** P<0.01; *** P <0.001; **** P<0.0001, (Student’s t-test). Blue asterisks indicate significant differences compared to WT and red asterisks indicate statistical significance between -/+ RNH conditions for each mutant. Average and SD of three independent experiments are shown. **C.** Left panel: Representative image of Mph1-GFP foci accumulating in *rnh1 rnh201* mutants. Arrowheads indicate foci. Scale bar, 3 μm. Right Panel: Mph1-GFP foci were quantified in exponentially growing wild type (ySLG636), *rnh1* (ySLG639), *rnh201* (ySLG640), and *rnh201 rnh1* (ySLG642) cells (OD_600_ = 0.3) at 25°C. Cells were divided into budded cells in the S or G2 phase of the cell cycle or into un-budded, G1-cells. Two replicates of 200–600 cells were examined. Error bars indicate 95% confidence intervals. Significance relative to the wild type determined by Fisher’s exact test (** P<0.05). **D.** The heterozygous diploid strain (ySLG605, BY4741 background) was sporulated and micromanipulated onto YPD agar plates to generate haploid offspring. Spores were incubated for 3 days at 30°C before imaging (left panel). **E.** yAL296 was arrested in G1 with α-factor and released into YPD media at 25°C. Protein extracts were prepared at the indicated time points (minutes post G1 release) and subject to western blotting. **F.** yAL404 was sporulated and dissected onto YPD media and images were acquired following 3 days of incubation at 30°C. Strains were genotyped by selectable markers and PCR techniques. **G.** The status of Rad53 phosphorylation (up-shift) was analyzed by western blotting with anti-Rad53 specific antibodies. Rnr3 protein levels were assessed by probing with an anti-Rnr3 antibody. Ponceau staining served as a loading control. **H.** Wild type **(**WT) (top) and *mph1 rnh1 rnh201* cells (bottom) were transformed with an Mph1 wild type expressing plasmid or with helicase-defective Mph1 variants (E210Q and Q603D). Serial dilutions were spotted onto selective media. Pictures were taken after growing cells for 3 days at 30°C.

Given the involvement of the human Fanconi Anemia M protein in preventing R-loop-mediated damage and RNA-DNA hybrid accumulation *in vivo*, we decided to address whether yeast cells deleted for the FANCM homolog *MPH1* or other candidate helicases implicated in DNA repair are also involved in R-loop metabolism. Deletion mutants of *SGS1*, *SRS2*, *MPH1*, *RRM3* were assayed for the accumulation of Rad52 foci, a marker for DNA repair at sites of damage. Genotypes were considered as specifically accumulating RNA-DNA hybrid-induced damage when the increased number of Rad52 foci was abolished upon RNase H1 overexpression. The deletion of the RNase H enzymes (*rnh1 rnh201*) and *HPR1*, a member of the THO complex, where R-loop accumulation has been demonstrated to account for the majority of DNA damage [9], served as positive controls. It is worth noticing that Rad52 foci were not fully suppressed in *rnh1 rnh2* cells, which suggests that hybrids not easily accessible to RNase H1 may accumulate in these double mutants. Although all four single gene mutants tested led to a significant increase in Rad52 foci compared to wild type, only *MPH1* deleted cells showed an RNase H1-sensitive increase (Fig 1A). Therefore, we focused on achieving a more complete understanding of Mph1 and its role at RNA-DNA hybrids. To rule out yeast genetic background specific effects, we confirmed the accumulation of Rad52 foci in *mph1* mutants in the W303 genetic background (Fig 1B). We performed a western blot on an endogenously tagged Rad52 strain (Rad52-TAP) with and without RNase H1 overexpression to rule out an effect on Rad52 protein levels (S1A Fig). This indicates that Mph1 has a role in the prevention of RNA-DNA hybrid formation or in minimizing the damage induced by RNA-DNA hybrids already formed.

In order to get an indication of whether Mph1 may be acting directly at RNA-DNA hybrids, we tested whether it forms foci in mutants that are known to accumulate hybrids. We observed an increased frequency of endogenously expressed Mph1-GFP foci in *rnh1 rnh201* double mutant cells, where hybrids accumulate to high levels (Fig 1C). Mph1-GFP foci specifically accumulated in *rnh1 rnh201* double mutant cells in the S- or G2 phase of the cell cycle and not in G1 (Fig 1C), suggesting that Mph1 plays a role at RNA-DNA hybrids during DNA replication to prevent the accumulation of DNA damage.

### *MPH1* is essential in the absence of RNase H1 and RNase H2

As Mph1 forms foci when RNA-DNA hybrids accumulate, we next investigated whether its function becomes essential when known RNA-DNA hybrid regulating factors are impaired. Indeed, it has been reported that the loss of *MPH1* leads to growth impairment in the absence of *RNH203* in the S288C background [32]. In the BY4741 and W303 genetic backgrounds, the *mph1 rnh1 rnh201* triple mutants were either severely growth compromised or completely inviable, respectively (Fig 1D and S1B Fig, for detailed growth curves and population doubling times see S1C Fig). In order to test if this essential function of Mph1 occurs during S phase, we created an allele of *RNH1* (*S*-*RNH1*) that is specifically expressed in S phase, by placing *RNH1-TAP* under the Clb6 promoter and fusing it to a Clb6 degron sequence [40] (Fig 1E, S1D Fig). Compared to the *rnh1 rnh201 mph1* triple mutant, the expression of Rnh1 in S phase did not have a growth defect in *rnh201 mph1* mutants (Fig 1F). Consistent with Mph1 preventing R-loop-induced damage, the slow growth of the *rnh1 rnh201 mph1* mutants was associated with a slightly increased phosphorylation of Rad53 as well as induced expression of *RNR3* (Fig 1G), both being established markers for activation of the DNA damage checkpoint. To test whether Mph1’s helicase activity is essential to restore the growth of *rnh1 rnh210 mph1* triple mutants, we reintroduced helicase-compromised Mph1 mutants, *mph1*-E210Q and *mph1*-Q603D [34], on a plasmid by expressing them from the endogenous *MPH1* promoter. Whereas wild type Mph1 fully suppressed the growth defect and checkpoint activation, neither of the helicase mutants was able to rescue the synthetic interaction (Fig 1H and S1E Fig), despite being expressed at similar levels (S1F Fig).

As RNase H2 activity is capable of removing single ribonucleotides that have been misincorporated into DNA helix by the replicating DNA polymerase [41, 42], as well as consecutive RNA-DNA hybrids (i.e. those formed in an R-loop), we wanted to determine at which type of RNA-DNA hybrid Mph1 was functioning. Therefore, we employed the *RNH201-P45D-Y219A* mutant, which is specifically defective in mono-ribonucleotide excision repair (RER), but is proficient in removing longer hybrid stretches (hereafter called *RNH201-RED* for ribonucleotide excision defective) [43, 44]. We transformed this plasmid into the *rnh1 rnh201 mph1* cells and observed that its expression complements the synthetic growth defect to the same extent as wild type *RNH201* (S1G Fig). Consistently, the checkpoint activation, as monitored by Rnr3 expression, was also alleviated upon expression of both wild type *RNH201* and *RNH201-RED* (S1H Fig). We verified that the *RNH201-RED* allele is RER-defective *in vivo* by demonstrating that the expression of this mutant could not suppress the hydroxyurea (HU) sensitivity of *pol2M644G rnh201* double mutants, which harbors high levels of misincorporated ribonucleotides [45], whereas wild type *RNH201* was able to complement (S1I Fig). In summary, these data indicate that Mph1’s helicase activity becomes essential during DNA replication when consecutive RNA-DNA hybrids (such as those present in R-loops) accumulate and may not be required at misincorporated ribonucleotide insertions.

**Fig 2:**
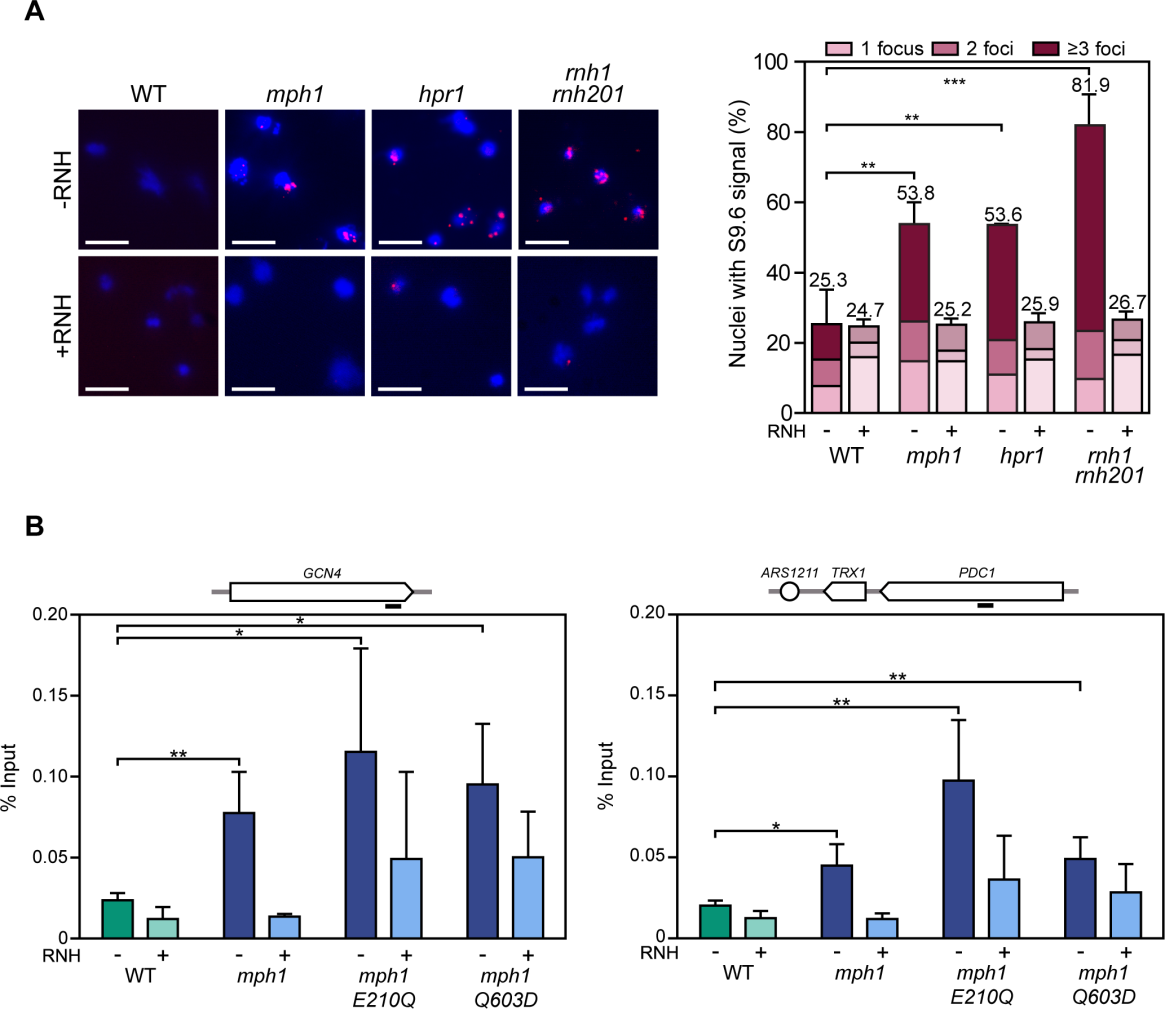
Accumulation of RNA-DNA hybrids in *mph1* cells depend on Mph1’s helicase domain. **A.** S9.6 foci detected by indirect immunofluorescence on yeast chromosome spreads in WT (W303.1A), *mph1* (WMPH1-2B*)*, *hpr1* (HPBAR1-R) and *rnh1 rnh201* (RNH-R*)* strains carrying the empty plasmid pCM189 (*-RNH*) or pCM189::RNH1 (*+RNH*). Representative spreads are shown, DNA is stained in blue (DAPI), red foci represent RNA-DNA hybrids detected by the S9.6 antibody. Scale bar = 5 μm (left panel). Quantification of nuclei containing S9.6 foci is shown. Data represent mean and SD of three to four independent experiments in which 150–200 nuclei were counted for each replicate. Statistical analysis was performed with the total number of signals for each mutant with respect to WT value (right panel). **B.** DRIP with S9.6 antibody in WT (W303-1A), *mph1* (WMPH1-2B), *mph1-*Q603D (T597-1) and *mph1-*E210Q (T617) strains in asynchronous cultures treated (+) or not (-) *in vitro* with RNase H in the *GCN4* and *PDC1* genes (left and right panel respectively). Data represent mean and SD of four independent experiments. * P<0.05; ** P<0.01; *** P<0.001 (two-tailed Student’s t-test). Black bar depicts the approximate primer binding region for amplification of the respective locus.

As Mph1’s activity is required in *rnh1 rnh201* double mutants we tested whether Mph1 is also essential in other mutants that have been reported to accumulate RNA-DNA hybrids. Unlike the loss of both RNase H1 and H2 functions, we found that deleting *MPH1* does not result in growth impairment when either of the THO components, *THP2* (S2A Fig) or *HPR1*, are deleted (S2B Fig), or in combination with the temperature-sensitive *sen1-1* allele (S2C Fig). Consistently, we also did not see evidence of DNA damage checkpoint activation as shown by both lack of detectable Rad53 phosphorylation and *RNR3* induction, in *mph1 hpr1* double mutants in the BY4741 background (S2D Fig). Nonetheless, Mph1 foci were observed in *hpr1* mutants and these foci were suppressed by RNase H1 overexpression (S2E Fig), consistent with the notion that Mph1 foci are enriched at R-loops. These results suggest that Mph1 may only become essential when RNA-DNA hybrids reach very high levels as in RNase H-deficient double mutant cells (see Fig 2A right). Alternatively, Mph1 may be required to act on a subset of RNA-DNA hybrids that is distinct from those affected by the THO and Sen1 proteins.

### Mph1 prevents the accumulation of RNA-DNA hybrids

The combined results of the synthetic growth defect between *mph1* and *rnh1 rnh201* mutants together with the fact that Rad52 foci accumulate in *mph1* cells in an RNase H1-sensitive manner led us to speculate that RNA-DNA hybrids may accumulate in *mph1* mutants. Since R-loops frequently lead to hyper-recombination, we sought to study recombination in the absence of Mph1. For that, we used previously devised direct-repeat recombination assays based on two truncated copies of the *LEU2* gene [46]. Unlike mutants of the THO complex or *sen1-1* mutants, where R-loops accumulate, deletion of *MPH1* results in only conservative changes, or no changes at all, in recombination, depending on the assay used (S3A Fig, S3B Fig). These data, together with the RNase H-sensitive increase of Rad52 foci (Fig 1A) suggest that in the absence of the Mph1 helicase activity, the damage caused by RNA-DNA hybrids is not efficiently resolved into detectable recombination products and hence results in the accumulation of Rad52 foci.

To assay for the presence of RNA-DNA hybrids, we performed indirect immunofluorescence on chromosome spreads. We detected an increase in the number cells with S9.6 foci in *mph1* mutant cells, indicating increased levels of RNA-DNA hybrids (Fig 2A). Cells containing more than 3 foci, were especially prevalent when *MPH1* was deleted. *hpr1* and *rnh1 rnh201* cells served as positive controls, where hybrids are known to accumulate (Fig 2A, right panel). To confirm the accumulation of RNA-DNA hybrids in *mph1* mutants we used DNA-RNA immunoprecipitation (DRIP) to pull down RNA-DNA hybrids as previously described [26, 47]. In agreement with the immunofluorescence data (Fig 2A), RNA-DNA hybrids accumulated significantly within the protein coding genes, *GCN4* (Fig 2B, left panel) and *PDC1* (right panel) in *mph1* mutants. The RNA-DNA hybrids signal could be strongly reduced upon *in vitro* RNase H1 treatment, thereby demonstrating the specificity of the DRIP signal. To investigate whether Mph1’s helicase activity is needed to prevent the accumulation of RNA-DNA hybrids, we also performed DRIP on helicase-dead Mph1 mutants (*mph1*-E210Q and *mph1*-Q603D) [36], which accumulated RNA-DNA hybrids at the *GCN4* (Fig 2B, left) and the *PDC1* (right) loci similarly to the complete deletion.

**Fig 3:**
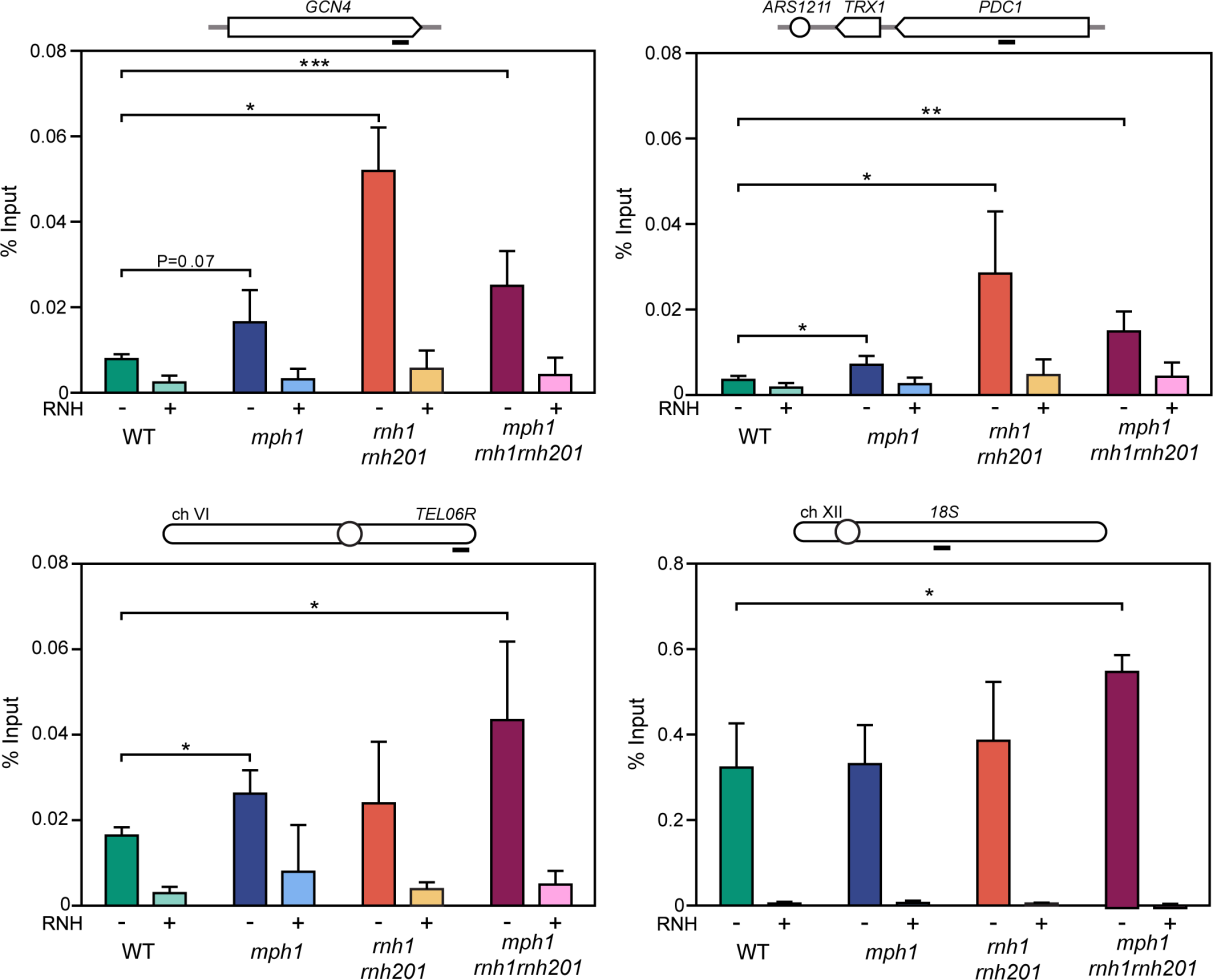
Accumulation of RNA-DNA hybrids in the triple mutant *mph1 rnh1 rnh201*. DRIP with the S9.6 antibody in WT (yBL7), *mph1* (ySLG2), *rnh1 rnh201* (ySLG428) and *mph1rnh1 rnh201* (ySLG611) strains in asynchronous cultures treated (+) or not (-) *in vitro* with RNase H in the *GCN4* and *PDC1* genes (top, left and right panels respectively) and TEL06R and rDNA18S loci (bottom, left and right panels respectively). Data represent mean and SD of four independent experiments. * P<0.05; ** P<0.01; *** P<0.001 (two-tailed Student’s t-test). Black bar depicts the approximate primer binding region for amplification of the respective loci.

To better understand how Mph1 is involved in RNA-DNA hybrid regulation, we expanded our DRIP analysis to telomeres (telomere 6R) and the *rDNA* loci (Fig 3). Whereas loss of Mph1 function did not affect hybrid levels at the 18s *rDNA* locus, it led to an increase at telomere 6R, suggesting that Mph1 may act preferentially at RNA polymerase II transcribed loci. When DRIP was performed in the *mph1 rnh1 rnh2* mutants (only viable in BY4741 background, Fig 1), we did not observe any further increase in RNA-DNA hybrids with respect to the single *mph1* or double *rnh1 rnh2* mutants (Fig 3). A similar epistasis was observed for Rad52 foci (Fig 1A), suggesting that Mph1 may remove RNA-DNA hybrids via RNase H.

### Mph1 accumulates at short telomeres in response to increased TERRA and R-loops

**Fig 4:**
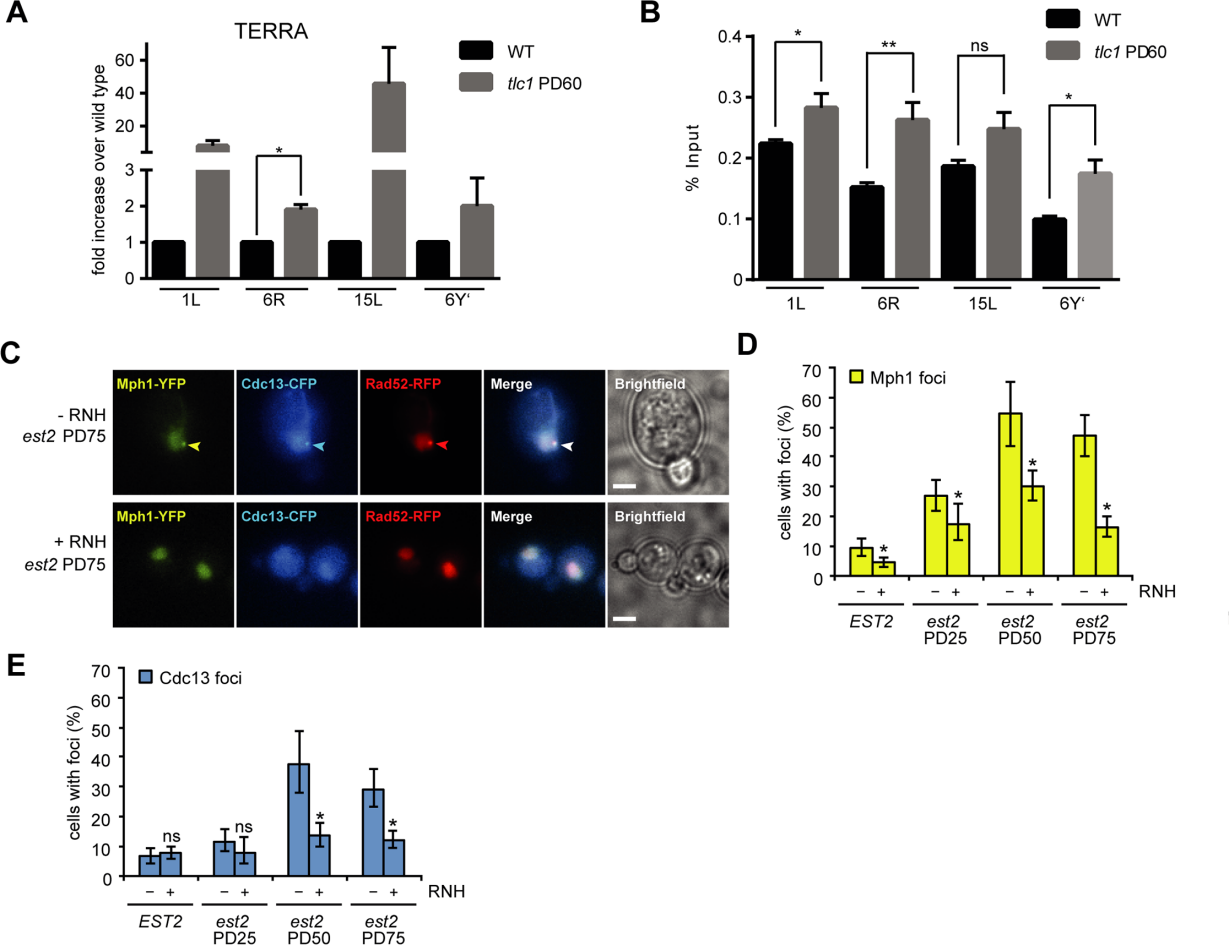
Mph1 forms foci at telomeres during senescence, when RNA-DNA hybrids accumulate. **A.** Both wild type and *tlc1* cells were derived from the *TLC1/tlc1* heterozygous diploid yAL95 and grown for approximately 60 population doublings (PD) before RNA was extracted from exponentially growing cells. Following reverse transcription with a telomeric sequence, TERRA levels were analysed at the indicated telomeres via qPCR with subtelomeric specific primer pairs (see methods and S1 Table for details). Three biological replicates were used for each genotype. Error bars indicate 95% confidence intervals. * represents significance relative to wild type determined by Student’s t-test (P<0.05). **B.** Both wild type and *tlc1* cells were grown for approximately 60 population doublings. ChIP with the S9.6 antibody that specifically recognizes RNA-DNA hybrids followed by qPCR. Error bars represent 95% confidence intervals, *, significance relative to wild type as determined by a Student’s t-test (P<0.05). **C.** Representative images for senescing cells showing co-localization between Mph1-YFP, Rad52-yEmRFP and Cdc13-CFP. Scale bar, 3 μm. Quantification of Mph1 (**D**.) and Cdc13-foci (**E**.). RNase H1 was expressed from a plasmid (pBB39).

The overexpression of Mph1 drastically increases the rate of replicative senescence in cells harboring short telomeres following the loss of telomerase activity [39], implying that it may be functionally relevant at telomeres. Levels of the non-coding telomeric repeat-containing RNA (TERRA) increase as telomeres shorten in yeast and human cells [22, 23, 48]. Moreover, RNA-DNA hybrids (presumably involving TERRA) can be detected at telomeres and accumulate in the absence of the RNase H enzymes [18–20, 49]. We allowed wild type and telomerase negative (*tlc1*) cells to undergo approximately 60 population doublings and, as expected, observed an increase in TERRA levels at all tested telomeres (Fig 4A). Upon performing DRIP on wild type and telomerase negative cells to monitor hybrid levels, we observed an increase in telomeric hybrids despite the fact that they were shorter (Fig 4B). Importantly, in an independent experiment, we observed that the overexpression of RNase H1 abolished the increased DRIP signal in *tlc1* cells (S4A Fig), demonstrating the specificity of the RNA-DNA hybrid signal shown in Fig 4B. To verify that Mph1 was acting at short telomeres in an RNA-DNA hybrid dependent manner, we monitored Mph1-YFP foci formation as telomeres shorten in telomerase negative cells (*est2*). Importantly, we observed that Mph1-YFP foci accumulate as telomeres shorten (Fig 4C and 4D). At the peak of senescence more than 50% of Mph1 foci co-localize with Cdc13 (which represent dysfunctional telomeres) and Rad52 foci, thereby indicating that they accumulate at critically short and dysfunctional telomeres (Fig 4C–4E) [50]. Importantly, Mph1’s ability to form foci was greatly reduced when RNase H1 was overexpressed (Fig 4C and 4D). Strikingly, the number of Cdc13 foci was also decreased upon RNase H overexpression, suggesting that hybrids may be a source of DNA damage at telomeres upon shortening. By ChIP analysis we could see that loss of the C-terminal domain of Mph1, which has been shown to interact with Rfa1 and Mte1 [30, 51], prevented Mph1 from localizing to telomeres in telomerase positive cells (S4B Fig) indicating that Mph1 location at telomeres may depend on its interaction with replication protein A (RPA). Taken together, we conclude that Mph1 accumulates at short telomeres, in an RNA-DNA hybrid-dependent manner.

### The Smc5/6 complex negatively regulates Mph1 at RNA-DNA hybrids

**Fig 5:**
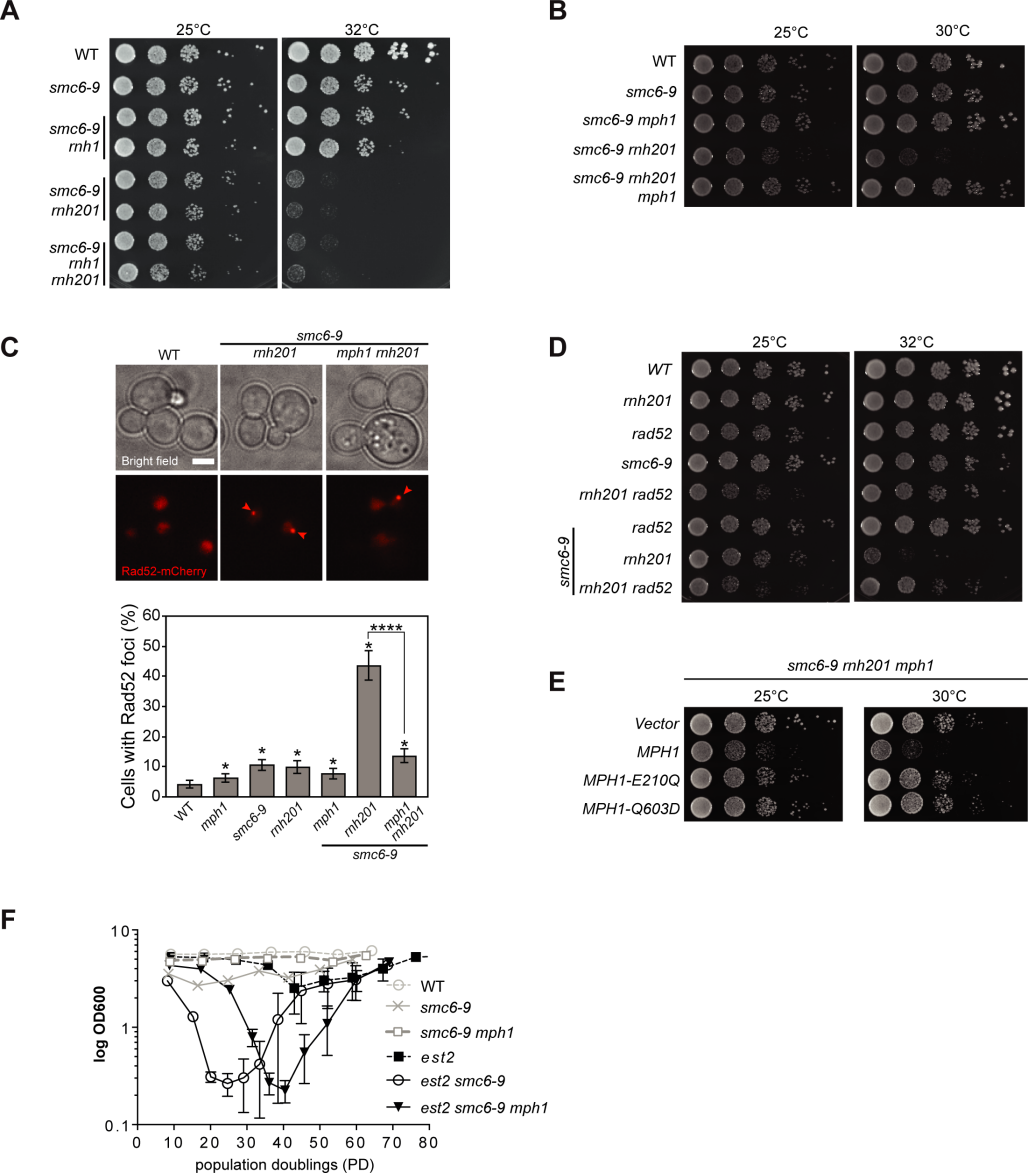
The SMC5/6 complex regulates Mph1 at RNA-DNA hybrids. **A.** Yeast haploid cells with the indicated genotypes were generated following tetrad dissection of ySLG419. Cells were grown overnight in liquid YPD at 23°C and spotted in 10-fold serial dilutions at the indicated temperatures on YPD agar. Digital images were acquired following 2 days of incubation. **B.** Haploids were derived from dissection of ySLG419 and ten-fold serial dilutions were spotted. Images were taken after 2 days. **C.** *MPH1*-dependent accumulation of Rad52 foci in *rnh201 smc6-9* mutants. Spontaneous Rad52-mCherry foci were quantified in wild type (yBL1052), *mph1* (YBL1051), *smc6-9* (yBL1047), *rnh201* (yBL1053), *mph1 smc6-9* (yBL1050), *rnh201 smc6-9* (yBL1048), and *mph1 smc6-9 rnh201* (yBL1049) after shifting an exponentially growing culture (OD_600_ = 0.3) from 25°C to 30°C for 3 hours in SC medium supplemented with 100 μg/ml adenine. Upper panel: Representative images of Rad52 foci. Arrowheads indicate foci. Scale bar, 3 μm. Lower panel: Quantification of Rad52 foci. Two replicates of 200–600 cells were examined. Error bars indicate 95% confidence intervals. *, significance relative to the wild type determined by Fisher’s exact test (P < 0.05). **D.** The heterozygous diploid strain yBL1022 was micro-dissected, and the haploid offsprings with the indicated genotype were spotted in 10-fold serial dilutions onto YPD-agar and incubated for 2 days at 30°C. **E.** *smc6-9 rnh201 mph1* was transformed with either empty vector or *MPH1* expression plasmids (*WT* allele or helicase-dead mutants). The transformants were spotted on selective media and grown for three days at 25°C or for two days at 30°C. **F.** The indicated genotypes were derived via tetrad dissection of ySLG115. Cells were diluted daily (every 24 hours) to OD_600_ 0.01 before the density was re-determined and cells were re-diluted. 6 biological replicates were used for every genotype indicated. Error bars represent SEM.

Previous data indicates that the Smc5/6 complex is important to limit the accumulation of toxic recombination intermediates in the presence of MMS (methyl methanesulfonate), and at natural pause sites [34, 35, 52–54]. A negative genetic interaction between the *smc6* mutants and *rnh201* as well as *rnh202* was previously described in a high-throughput SGA screen [55]. Furthermore, there is ample evidence that the Smc5/6 complex is important for the accurate replication of the rDNA locus, a repetitive genomic region rich in RNA-DNA hybrids [56, 57]. Based on these observations, we hypothesized that the Smc5/6 complex might be required for the accurate processing of DNA replication intermediates that arise when RNA-DNA hybrids are encountered by the replisome. To test this notion, we introduced the *smc6-9* and *smc5-6* temperature sensitive alleles into strains defective for either RNase H1 (*rnh1*) or RNase H2 (*rnh201*) activity, as well as in *rnh1 rnh201* double mutants. The absence of *RNH201*, but not *RNH1*, resulted in a severe growth defect, when either *SMC6* (Fig 5A) or *SMC5* (S5A Fig) function was reduced.

Next, we tested whether the deletion of *MPH1* would rescue the above described synthetic growth defect between *smc5/6* mutants and *rnh201*. We observed that growth defects of both *smc6-9 rnh201* and *smc5-6 rnh201* cells were alleviated in the absence of *MPH1* (Fig 5B, and S5B Fig). In accordance with the synthetic growth defect occurring as a result of replication stress, we observed that *smc6-9 rnh201* cultures accumulated cells with a 2N DNA content in comparison to both wild type and the respective single mutants (S5C Fig). Importantly, the accumulation of cells with a 2N DNA content was reversed when *MPH1* was additionally deleted (S5C Fig).

In order to confirm that DNA damage accumulated in *smc6-9 rnh201* cells, we followed the formation of Rad52-mCherry foci (Fig 5C). We observed a dramatic increase in the number of cells with Rad52-mCherry foci in *smc6-9 rnh201* double mutants when compared to wild type and single mutant cells grown at semi-permissive temperature (Fig 5C, bottom panel). Strikingly, the deletion of *MPH1* reverted this accumulation to the levels observed in the respective single mutants (Fig 5C). We speculated that Mph1-dependent recombination intermediates accumulate and may be contributing to cellular toxicity. Indeed, upon deleting either the recombination factors *RAD52* or *RAD51* in *smc6-9 rnh201* mutants we observed an increased viability in the triple mutants as compared to the respective double mutants in a similar manner as to when *MPH1* was deleted (Fig 5D and S5D Fig). While the re-introduction of full-length *MPH1* led to a severe growth defect in *smc6-9 rnh201 mph1* cells, *mph1-E210Q* and *mph1-Q603D* helicase mutants behaved similar to the complete deletion of *MPH1* (Fig 5E). Moreover, introduction of the *RNH201-RED* allele complemented the synthetic growth defect, nearly to the same extent as wild type *RNH201* (S5E Fig). The overexpression of RNase H1, was not able to alleviate the growth defects of the double mutants, which may indicate an RNase H2 specific sub-set of RNA-DNA hybrids that lead to toxicity (S5F Fig). Finally, the deletion of *RNH202*, an auxiliary component of RNase H2 also showed negative growth defects when combined with *smc6-9* (S5G Fig). A plasmid harboring full-length *RNH202* complemented the growth defect as did an *RNH202-ΔPIP* mutant, where the proliferating cell nuclear antigen (PCNA) interaction motif had been mutated [43](S5G Fig). These data strongly suggest that the helicase activity of Mph1 needs to be counteracted by the Smc5/6 complex at consecutive RNA-DNA hybrids, and to a lesser extent at misincorporated ribonucleotides, in order to prevent the accumulation of toxic recombination products.

Finally, we investigated whether the Smc5/6-mediated negative regulation of Mph1 function at RNA-DNA hybrids may also be conserved at short telomeres, where hybrids accumulate (Fig 4B). Interestingly, Smc5/6 has recently been demonstrated to regulate TERRA levels [58] and we observed that Mph1 suppresses the accumulation of RNA-DNA hybrids at telomere 6R (Fig 3). In the absence of a functional Smc5/6 complex, telomerase negative cells (*est2*) lose growth potential at very early population doublings (Fig 5F) consistent with the previously reported premature senescence phenotype [37, 38]. Importantly, the additional loss of *MPH1* alleviated, to a great extent, the premature senescence of *smc6-9 est2* cells (Fig 5F). These data led us to the conclusion that the Smc5/6 complex may be required to limit or process Mph1-mediated intermediates at short telomeres harboring RNA-DNA hybrids. Importantly, we did not detect increased levels of R-loops at either the *GCN4* or *PDC1* loci when either *SMC5* or *SMC6* were inactivated (S5H Fig).

In summary, our combined observations suggest that while Mph1 activity is required during replication through RNA-DNA hybrids, its unrestrained helicase activity may lead to the formation of lethal recombination intermediates at RNA-DNA hybrids. However, this toxic activity can be prevented by the presence of a functional Smc5/6 complex.

## Discussion

We set out to identify additional helicases that are involved in RNA-DNA hybrid metabolism through our small-scale candidate genetic screen looking for mutants with an accumulation of DNA damage (Rad52 foci) that is abolished by overexpression of RNase H1. Here we identified Mph1 as a factor preventing DNA damage at RNA-DNA hybrids. We analyzed the effects of Mph1 in RNA-DNA hybrid turnover in more detail and found that Mph1 not only forms foci when RNA-DNA hybrids accumulate, but is also instrumental in preventing RNA-DNA hybrids from accumulating. Furthermore, we observed that *mph1 rnh1 rnh201* triple mutants are severely compromised for growth (Fig 1D), pointing towards Mph1 acting in either an alternative and/or complementary pathway to RNase H1 and RNase H2. The fact that Mph1 foci in *rnh1 rnh201* cells specifically occurred in S/G2, but not in G1 cells (Fig 1C) suggests that Mph1 plays a role at RNA-DNA hybrids after they are encountered by a replication fork. This is further supported by the fact that the expression of *RNH1* specifically in S phase, is sufficient to reverse the lethality of *rnh1 rnh201 mph1* mutants (Fig 1F). Taking the *in vitro* enzymatic activities into account, we speculate that Mph1 may promote fork reversal (see below), which can contribute to fork restart [59], when replication forks stall at RNA-DNA hybrids. Restart of replication forks is especially crucial when DNA replication proceeds unidirectionally, i.e. at the telomere or in the rDNA, because converging replisomes do not exist to complete replication. In this regard, it is interesting that the SMC5/6 complex, the negative regulator of Mph1, has been proposed to play a particularly important role at sites of unidirectional replication [36].

We report that mutants of the SMC5/6 complex show a synthetic growth defect with *rnh201* and *rnh202*, but not with *rnh1* mutants. The synthetic growth defect can be rescued by either impairing homologous recombination or by deleting *MPH1*. Interestingly, Rnh202, as well as its human homolog, are non-catalytic subunits of RNase H2 that contain a conserved PIP-box motif (a PCNA-interacting motif) that may account for RNase H activity at the replication fork in human cells [60]. RNase H1, in contrast, was not found to localize to replication foci during unperturbed replication. It is therefore hypothesized that when the replicative helicase encounters an RNA-DNA hybrid, PCNA provides a platform to recruit RNase H2 to the replication fork via the PIP-box motif. However, for the yeast RNase H2 PIP-box mutant no phenotype has been observed so far [43]. Moreover, we could rescue the growth defect of *smc6-9 rnh202* mutants by introducing the PIP-box defective RNase H2 allele (S5G Fig). This suggests that in yeast, RNase H2 may be recruited to the replication and repair machinery via a PCNA-independent mechanism.

**Fig 6:**
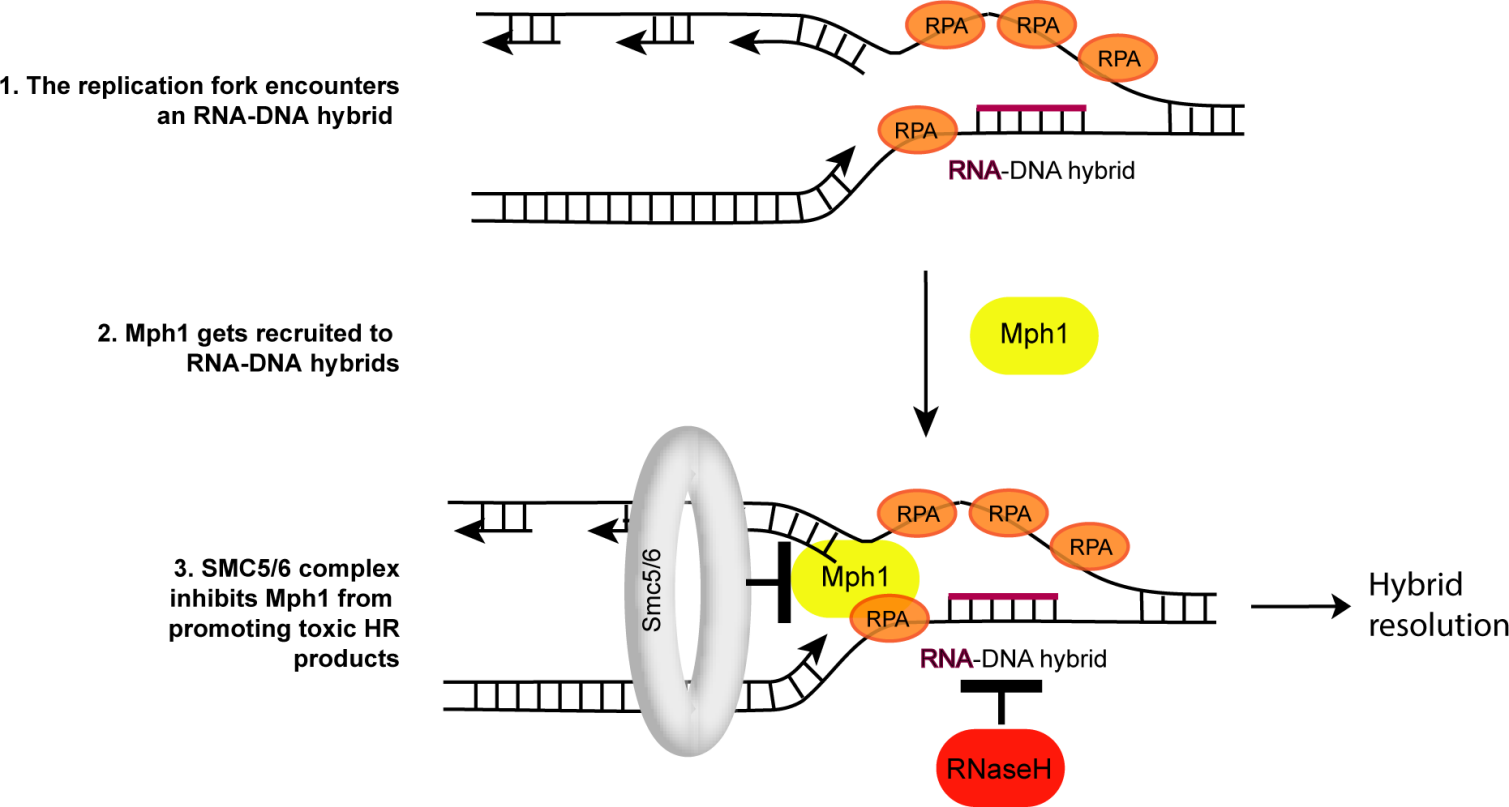
Mph1’s fork reversal activity might allow dissolution of RNA-DNA hybrids. When replication forks are paused at an RNA-DNA hybrid (either an R-loop as depicted, or consecutively incorporated ribonucleotides), Mph1 gets recruited to the stalled fork, potentially through its C-terminal interaction domain with RPA. Mph1 may directly remove the RNA-DNA hybrid (in the case of an R-loop) through its helicase activity, or promote the resolution by other factors (e.g. the RNase H enzymes as depicted). The Smc5/6 complex negatively regulates Mph1’s pro-recombinogenic activity at RNA-DNA hybrids to prevent toxic recombination intermediates from accumulating (see text for detailed explanation). Persistent R-loops at shortened telomeres may represent a natural scenario where such a regulation occurs.

Mph1 is able to promote helicase-dependent replication fork reversal *in vitro* [29, 61]. More recently the Smc5/6 complex has been implicated in specifically inhibiting the fork reversal activity of Mph1 *in vitro* [29], while not perturbing its D-loop remodeling capacities. The genetic data presented here, i.e. mutations in the inhibitory Smc5/6 complex being toxic in RNase H2 mutants and that this growth defect can be rescued by deleting *MPH1*, underlines the crucial regulation of Mph1 at sites of RNA-DNA hybrids, probably in the context of a replication fork. These results, together with previously published data have allowed us to propose a working model with respect to how Mph1 could act at replication forks stalled by RNA-DNA hybrids (Fig 6). When the replication fork encounters unresolved RNA-DNA hybrids, replication stress and fork stalling ensue (Fig 6-1). Either the accumulation of ssDNA at stalled forks or the ssDNA on the displaced strand of the R-loop may lead to the recruitment of Mph1, which can interact with the ssDNA binding complex, RPA (Fig 6-2). This is in line with the recent observation that RPA is implicated in R-loop metabolism *in vivo* and subsequent stimulation of RNase H1 activity *in vitro* [62]. Consistently, we observed an increase in the number of cells with Mph1 foci when RNA-DNA hybrids accumulated in *rnh1 rnh201* and *hpr1* mutants. Moreover, shortened telomeres, which accumulate RNA-DNA hybrids, associated more frequently with Mph1 foci in an RNase H sensitive manner (Fig 4C–4E). We hypothesize that the recruited Mph1 then remodels replication forks stalled at RNA-DNA hybrids to promote the subsequent removal of R-loops; perhaps via RNase H activity (Fig 6-3). The interaction with RNase H is supported by the genetic interactions between loss of *MPH1* and RNase H functions, e.g. epistatic interactions in terms of Rad52-GFP foci (Fig 1A) and R-loop accumulation (Fig 3). Finally, the Mph1-dependent synthetic lethality between *smc6-9* mutants and *rnh201* support the notion that the Smc5/6 complex must be present to ensure that Mph1 does not create toxic homologous recombination (Fig 5D) intermediates, possibly due to uncontrolled Mph1 fork reversal at R-loops (Fig 6-3).

Recently, it has been shown that FANCM, the Mph1 homolog in higher eukaryotes, is able to branch migrate and thereby unwind R-loop structures *in vitro* [27]. Moreover, chicken DT40 cells lacking a functional FANCM translocase domain accumulate RNA-DNA hybrids [27]. Although we cannot exclude that Mph1 may directly unwind R-loop structures *in vivo*, we favor a role for Mph1 at the replication fork, based on its biochemical properties and our genetic studies which implicate a replication role (Fig 1C, 1E and 1F). Indeed, Mph1 recruitment to telomeres requires its C-terminal domain (S4B Fig), which has been shown to interact with RPA and Mte1. RPA specifically binds to ssDNA that has been unwound at the replication fork and Mte1 binds to branched DNA structures and recruits Mph1 to foci in S/G2 phase [63]. However, unlike deletion of *MPH1*, deletion of *MTE1* in *smc6-9 rnh201* cells did not alleviate the growth defects of the double mutant (S4C Fig). Thus, we conclude that Mte1 does not promote Mph1’s toxic activity at RNA-DNA hybrids in the absence of the Smc5/6 complex. Secondly, we hypothesize that if Mph1 were to unwind RNA-DNA hybrids in a more general manner, we would expect Mph1 to also be essential in *sen1-1* and THO mutants, but we did not see such genetic interactions. Finally, we could only detect Mph1 foci in S/G2 phase cells and not in G1 cells, suggesting that Mph1 is acting at replication forks and not at RNA-DNA hybrids *per se* (Fig 1C). Moreover, the S-Rnh1 allele alleviated the synthetic growth defect of *rnh1 rnh201 mph1* mutants (Fig 1F).

Therefore, Mph1 appears to work as a double-edged sword at RNA-DNA hybrids. Whereas Mph1 gets recruited into foci in a hybrid dependent manner and can promote the resolution of R-loops, its helicase activity must be controlled (by the Smc5/6 complex) to avoid toxic recombination intermediates. This interpretation is strongly supported by the fact that Mph1 becomes essential in *rnh1 rnh201* double mutants, but leads to lethality when RNA-DNA hybrids accumulate (loss of *RNH201*) in the absence of Smc5/6 function. Interestingly, the depletion of FANCM in human cells leads to replication forks travelling faster over small distances [64], but not when followed over longer time. This implies more replication fork pausing in the absence of FANCM. FANCM’s ATPase activity was necessary for slowing the replication fork and could therefore be a way of clearing RNA-DNA hybrids at the fork to ensure processive replication. Consistently, when the cells were challenged with CPT, which has been shown to lead to the accumulation of RNA-DNA hybrids [65, 66], FANCM was essential for replication fork progression and fork restart [64].

In summary, we have demonstrated that Mph1 and its regulation by Smc5/6 are critical at RNA-DNA hybrids. Further work is needed to determine whether Mph1 leads to fork reversal when the replication machinery is halted at RNA-DNA hybrids, and how Smc5/6 acts to prevent toxicity caused by Mph1 action on R loops. It will also be interesting to further understand how hybrids in different genomic contexts may require specific factors to ensure they are properly processed. This will especially be important in the context of diseases associated with faulty RNA-DNA hybrid processing such as AGS, ALS and AOA2.

## Materials and methods

### Yeast strains and plasmids

Standard procedures for yeast strain construction and cell growth were used [67]. Strains used for microscopy are *ADE2 LYS2 trp1-1* derivatives of W1588-4C [68], a *RAD5* derivate of W303-1A (*MAT***a** *ade2-1 can1-100 ura3-1 his3-11*,*15 leu2-3*,*112 trp1-1 rad5-535*) [69]. CFP-tagged Mph1 was generated for expression from its native chromosomal locus with a 4-alanine linker as described [70, 71] using primers listed in S1 Table and verified by sequencing. The *HPR1* gene was deleted with *KANMX6* in this strain to construct YBG722. Other strains are derivatives of BY4741 (*MAT***a** *his3*Δ*0 ura3*Δ*0 leu2*Δ*0 met15*Δ*0*). The construction of *RNH201 P45D Y219A*, was described previously [25]. A complete list of strains and plasmids used in this study are listed in S2 Table and S3 Table, respectively.

### Microscopy and immunofluorescence

Yeast cells were grown and processed for fluorescence microscopy as described previously [72]. Fluorophores were cyan fluorescent protein (CFP, clone W7) [73], yellow fluorescent protein (YFP, clone 10C) [74] and red fluorescent protein (RFP, clone yEmRFP; or mCherry) [75]. Fluorophores were visualized on a Deltavision Elite microscope (Applied Precision, Inc) equipped with a 100x objective lens (Olympus U-PLAN S-APO, NA 1.4), a cooled Evolve 512 EMCCD camera (Photometrics, Japan), and an Insight solid-state illumination source (Applied Precision, Inc). Pictures were processed with Volocity software (PerkinElmer). Images were acquired using softWoRx (Applied Precision, Inc) software.

Spontaneous Rad52-YFP foci from mid-log growing cells carrying plasmid pWJ1344 were visualized and counted by fluorescence microscopy as described in [76].

### Recombination frequency assays

For the recombination assays, cells were cultured in SC medium plates and grown for 3 to 4 days. Leu+ recombinants resulting from recombination in LYΔNS and TL-lacZ systems were selected on SC-Leu plates. Recombination frequencies were obtained by fluctuation tests as the median value of six independent colonies isolated from SC medium plates. The final frequency given for each strain and condition is the mean and SD of at least three median values, as described previously [77].

### Western blot

Proteins were extracted and analysed via western blotting according to Klermund et al, 2014 [78]. The Rad53 antibody (EL7.E1; gift from M. Foiani) was used in a 1:16 dilution; the HA antibody (clone 16B12; covance) was used in a 1:2000 dilution. Anti-RNR3 is a polyclonal antibody from Agrisera antibodies and was used as a 1:300 dilution.

### Chromatin immunoprecipitation

RNA-DNA hybrid analysis (for Fig 4B) as well as protein chromatin immunoprecipitations at telomeres were performed as previously published [20]. Mph1-Myc protein was immunoprecipitated with an antibody against the Myc tag (clone 9B11, Cell Signaling/ NEB). Otherwise the protocol did not differ from the standard ChIP protocol referenced above. The following primers (for sequence see S1 Table) were used during the quantitative PCR step: oBL295 and oBL296 (500 nM final) to amplify the 1L telomere, oLK57 and 58 (100 nM) target the 15L telomere and oLK49 and oLK50 (300 nM) are specific for the 6Y’ telomeres.

### DRIP and qPCR

Mid-log cultures grown in YPAD at 30°C or 32°C (for temperature sensitive strains) were collected. RNA-DNA hybrids were processed and analyzed as described [47]. For the negative control, half of the DNA was treated with 8μl RNase H (New England BioLabs) overnight at 37°C. Quantitative PCR was performed at the indicated regions using the SYBR Green PCR Master Mix (Biorad) and a 7500 Fast Real Time PCR System (Applied Biosystems). The relative abundance of RNA-DNA hybrid immunoprecipitated in each region was normalized to the signal obtained in the inputs. Average and standard deviation of at least three independent experiments are shown.

### Chromosome spreads and S9.6 immunofluorescence detection

Chromosome spread from cells grown to mid-log phase in YPAD were prepared and labeled with the monoclonal antibody S9.6 and immunodetected with Cy3- conjugated goat anti-mouse antibody (Jackson Laboratories, #115-165-003 as described [79]). Slides were mounted with 50 μl of VectaShield (Vector Laboratories, CA) with 1x DAPI and sealed with nail polish. For each replicate (n>3), between 150 and 250 nuclei were visualized and manually counted to obtain the fraction with detectable RNA-DNA hybrids.

### Replicative senescence assay

For Mph1-foci quantification, cells with *EST2* deleted in the genome were propagated at 25°C in liquid dropout medium lacking uracil to preserve the plasmid pAP81 (for ectopic expression of *EST2*) and determine foci number prior to telomerase loss for Mph1-YFP, Cdc13-CFP and Rad52-RFP in the strain SS283-23D. Senescence was induced by streaking cells on solid YPD medium and checking for loss of growth on SC-Ura. Selected colonies were inoculated in liquid synthetic complete medium supplemented with 100 μg/ml adenine (SC+Ade) and propagated at 25°C for approximately 100 more population doublings. We estimate this procedure involved approximately 30–50 population doublings from the point of colony formation on solid YPD until cells were analyzed by microscopy for the first time point. Samples were collected for monitoring population doubling time by measuring OD_600_ and for live cell microscopy analysis at regular intervals. Cell cultures were kept at OD_600_ between 0.2 and 0.9 through the course of the experiment.

The senescence assay for the *smc6-9 est2 mph1* mutant experiment was performed as published previously [39].

### RNA extraction and TERRA level quantification

15 mL yeast cultures were grown to an OD_600_ of 0.6–0.8 and RNA was extracted via the hot phenol method [80]. Reverse transcription and quantitative real time PCR to determine TERRA levels were performed as previously described [81].

### Statistical analysis

For live cell microscopy experiments, the significance of the differences observed among cell populations was determined using one-tailed Fisher’s exact test. P-values with P < 0.05 were considered significant. Statistical analysis for the chromatin immunoprecipitation experiments was described previously [20]. Statistical tests (Student’s t-test and Mann-Whitney *U-test*) were calculated using GraphPad Prism software. In general, a *P*-value < 0.05 was considered statistically significant.

## Supporting information

S1 Fig **A.** Cells of the indicated genotype were grown to exponential phase in the presence or absence of RNase H1 overexpression and western blots were performed with the indicated antibodies. **B.** W303 strains *rnh1 mph1* (Ybpr1m1.1) and *rnh1 rnh201* (rnh1rnh201.8D) were crossed, sporulated and micromanipulated onto YPD agar plates. Picture was taken after 3 days of incubation at 30°C. The triple mutant *mph1 rnh1 rnh201* is lethal in the W303 genetic background. **C.** Upper: Growth curves of displayed mutants in YPD (BY4741 background). Cells were diluted to an OD_600_ of 0.05 in both technical and biological triplicates on a 96-well-plate. Cultures were incubated at 30°C and every hour cell density was determined. The mean together with the SEM are shown. n = 3. Lower: Population doubling time (PDT) is only impaired in *mph1 rnh1 rnh201*. The PDT for each curve was calculated in the exponential phase and the average PDT together with the SEM is displayed. P-values were calculated with Student’s t-test. n = 3 **D.** yAL296 was synchronized in G1 with α-factor and released into YPD media at 25°C. Flow cytometry to display DNA content is depicted. (exp = exponential phase, and time (min) after α-factor is indicated. **E.** Anti-Rnr3 Western blot to monitor DNA damage response or replication stress in the same genotypes as mentioned in (Fig 1H). Ponceau staining was used as a loading control. ‘Load’ refers to an unspecific band that arises due to cross-reaction with the Rnr3 antibody. **F.** Anti-HA Western Blot to control the protein expression level of HA-tagged Mph1 and its helicase mutants expressed from a plasmid (pBL301: vector control; pBL472-474: *MPH1*-1xHA; *mph1*-E210Q-1xHA; *mph1*-Q603D-1xHA, respectively). Ponceau staining of the membrane was used as loading control. **G.** Serial dilutions of wild type yeast cells (top) or *mph1 rnh1 rnh201* mutant cells (bottom) carrying either an empty centromeric plasmid (pBL97), a plasmid encoding for the wild type *RNH201* (pBL401) gene or the mutant *RNH201-RED(P45D-Y219A)* (pBL399). Cells were grown at 30°C and pictures were taken after 2 days. **H.** Anti-Rnr3 Western blot to monitor DNA damage response or replication stress in the same genotypes as mentioned in (G). Ponceau staining was used as a loading control. **I.** The *pol2M644G rnh201* double mutant was transformed with either empty vector, a plasmid carrying the *RNH201* wild type or the *RNH201-RED* allele and spotted on SD-URA plates containing the indicated concentrations of HU. The cells were grown for 48 hours at 30°C. The *rad53-11* mutant was used as a control strain that is HU sensitive.
(TIF)

S2 Fig **A.** The heterozygous diploid *THP2 MPH1/thp2 mph1* strain (YSLG649, left) was sporulated, micromanipulated and pictures were taken after two days incubation at 30°C. **B**. Tetrad analysis from crosses between *mph1* (WMPH1-2B) and *hpr1* (HPBAR-R1). Pictures were taken 4 days after micromanipulation at 30°C. **C.** Tetrad analysis of an *mph1 sen1-1* mutant cross (yMG187). Pictures were taken after 3 days incubation at 28°C. **D.** anti-Rad53 and -Rnr3 Western Blot in three different *hpr1 mph1* double mutants along with the single mutant controls. Treatment with HU served as positive controls for Rad53 phosphorylation and Rnr3 induction. Ponceau staining was used as loading control. **E.** Mph1-YFP foci accumulate in *hpr1* cells as determined by microscopy in WT (MLL66-11A) and *hpr1* (YBG722) cells following *RNH1* overexpression (pCM189::*RNH1*). Left panel: representative image with arrowheads pointing to Mph1 foci; right panel: quantification. (*) P < 0.05; (**) P<0.01 (Student’s t-test).
(TIF)

S3 Fig **A.** Recombination analysis of WT (W303-1A) and *mph1* (WMPH1-2B) strains carrying the TL-lacZ plasmid system, whose transcription is regulated by the *tet* promoter, in the presence (low transcription) or absence (high transcription) of 5 mg/mL doxycycline. **B.** Recombination analysis of WT (W303.1A), *mph1* (WMPH1.2B), *hpr1* (HPBAR-R1), *hpr1 mph1* (WMPHP.5A), *sen1-1* (SEN1-R), *sen1-1mph1* (WMPSEN.1C) strains carrying the LYΔNS plasmid. Leu+ recombinants resulting from recombination in TL-lacZ and LYΔNS systems were selected on SC-Leu plates. Mean and SD for at least three fluctuation tests consisting in the median value of six independent colonies each are shown. (*), P < 0.05, (**) P<0.01 (Student’s t-test). A scheme of the recombination system is shown on top.
(TIF)

S4 Fig **A.** DRIP was performed with the S9.6 antibody in order to determine the RNA-DNA hybrid levels in senescing cells (PD60 *tlc1*) in the presence or absence of Rnh1. *RNH1* was expressed endogenously under a galactose-inducible promoter (haploids derived from yMG103). Three different telomeres were analysed (1L, 6R and 6Y’). Individual qPCR data points are represented along with the mean (bar). **B.** Non-tagged wild type, Mph1-13myc (ySLG295) and Mph1^1-933^-13myc (ySLG527) were grown to exponential phase in liquid YPD media at 30°C. Cells were cross-linked and ChIP was performed using anti-myc monoclonal antibodies. ChIP signals were quantified by qPCR specific to 15L, 1L and 6Y’ telomeres. Error bars represent 95% confidence intervals where n = 3 biological isolates were used for each indicated genotype. (p-values were calculated by a student’s test; * p<0.05, ** p<0.005). **C.** yAL331 was sporulated and the indicated meiotic products with the corresponding genotypes were spotted at the indicated temperatures on YPD and imaged following 48 h incubation.
(TIF)

S5 Fig **A.** ySLG418 heterozygous diploids were microdissected and haploid offsprings were grown overnight in liquid YPD at 25°C and spotted as 10-fold serial dilutions onto YPD-agar at the indicated temperatures. Images were obtained after 2 days of incubation. **B.** The heterozygous diploid ySLG418 was microdissected to gain haploid offsprings. 10-fold serial dilutions were spotted onto YPD-agar at the indicated temperatures. **C.** DNA staining with Sytox Green coupled to flow cytometry was used to analyze the proportion of cells with a 1N and 2N DNA content. **D.** Yeast strains with the indicated genotypes were spotted as 10-fold serial dilutions onto YPD agar and incubated at the indicated temperatures following overnight growth in liquid YPD at 25°C. Images were acquired after 2 days of incubation. Haploids were derived from the heterozygous diploid strain yBL1021. **E.** *smc6-9 rnh201* (upper panel) and *smc5-6 rnh201* (lower panel) cells were transformed with an empty vector, or with plasmids containing either the *RNH201* wild type or the *RNH201-RED* allele. Expression of both alleles alleviated the growth defects at 32°C. Cells were grown at the indicated temperatures for two days. **F.** A plasmid expressing *RNH1* (pBB39) or an empty vector (pBL189) control were introduced into wild type, *smc6-9* and *smc6-9 rnh201* cells. The strains were grown at the indicated temperature for two days. **G.** *RNH202* wild type (pBL506) and *RNH202* lacking the PIP-box (pBL507) domain (*RNH202 ΔPIP*) were expressed from a plasmid in *smc6-9 rnh202* cells. Cells were grown for 72 hours at the indicated temperatures. *smc6-9 rnh202* cells carrying the empty vector (pBL505) served as control. **H.** DRIP with S9.6 antibody in WT (yBL7), *rnh201* (yBL435), *smc6-9* (ySLG88), smc5-6 (ySLG90) and *rnh201 smc6-9* (ySLG397) strains in asynchronous cultures treated (+) or not (-) *in vitro* with RNase H in the *GCN4* and *PDC1* genes (left and right panels respectively). Data represent mean and SD of five independent experiments. * P < 0.05; (two-tailed Student’s t-test). Black bar depicts the approximate primer binding region for amplification of the respective locus.
(TIF)

S1 Table Oligonucleotide primers. This table lists the primers used in this study.
(DOCX)

S2 Table Yeast strains. This table lists the yeast strains employed in this study.
(DOCX)

S3 Table Plasmid list. Plasmids used during this analysis are summarized in S3 Table.
(DOCX)
