# Supplementary figures and images for: The Smc5/6 complex regulates the yeast Mph1 helicase at RNA-DNA hybrid-mediated DNA damage
Source: PLoS Genet. 2017 Dec 27;13(12):e1007136. doi: 10.1371/journal.pgen.1007136 (PMC5760084; doi:10.1371/journal.pgen.1007136)

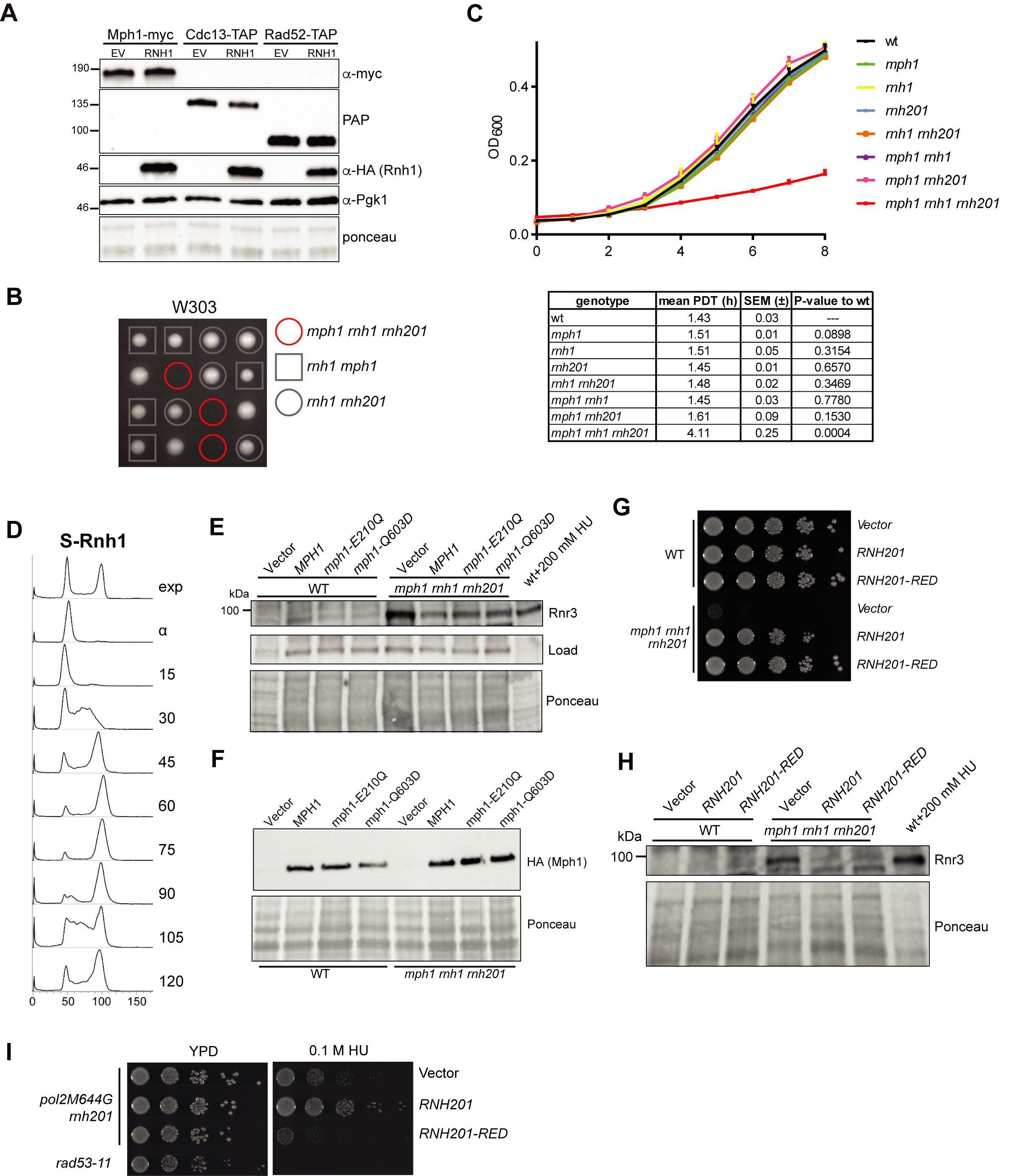

Supplement: S1 Fig — A. Cells of the indicated genotype were grown to exponential phase in the presence or absence of RNase H1 overexpression and western blots were performed with the indicated antibodies. B. W303 strains rnh1 mph1 (Ybpr1m1.1) and rnh1 rnh201 (rnh1rnh201.8D) were crossed, sporulated and micromanipulated onto YPD agar plates. Picture was taken after 3 days of incubation at 30°C. The triple mutant mph1 rnh1 rnh201 is lethal in the W303 genetic background. C. Upper: Growth curves of displayed mutants in YPD (BY4741 background). Cells were diluted to an OD600 of 0.05 in both technical and biological triplicates on a 96-well-plate. Cultures were incubated at 30°C and every hour cell density was determined. The mean together with the SEM are shown. n = 3. Lower: Population doubling time (PDT) is only impaired in mph1 rnh1 rnh201. The PDT for each curve was calculated in the exponential phase and the average PDT together with the SEM is displayed. P-values were calculated with Student’s t-test. n = 3 D. yAL296 was synchronized in G1 with α-factor and released into YPD media at 25°C. Flow cytometry to display DNA content is depicted. (exp = exponential phase, and time (min) after α-factor is indicated. E. Anti-Rnr3 Western blot to monitor DNA damage response or replication stress in the same genotypes as mentioned in (Fig 1H). Ponceau staining was used as a loading control. ‘Load’ refers to an unspecific band that arises due to cross-reaction with the Rnr3 antibody. F. Anti-HA Western Blot to control the protein expression level of HA-tagged Mph1 and its helicase mutants expressed from a plasmid (pBL301: vector control; pBL472-474: MPH1-1xHA; mph1-E210Q-1xHA; mph1-Q603D-1xHA, respectively). Ponceau staining of the membrane was used as loading control. G. Serial dilutions of wild type yeast cells (top) or mph1 rnh1 rnh201 mutant cells (bottom) carrying either an empty centromeric plasmid (pBL97), a plasmid encoding for the wild type RNH201 (pBL401) gene or the mutant RNH201-RED( [file pgen.1007136.s001.tif]

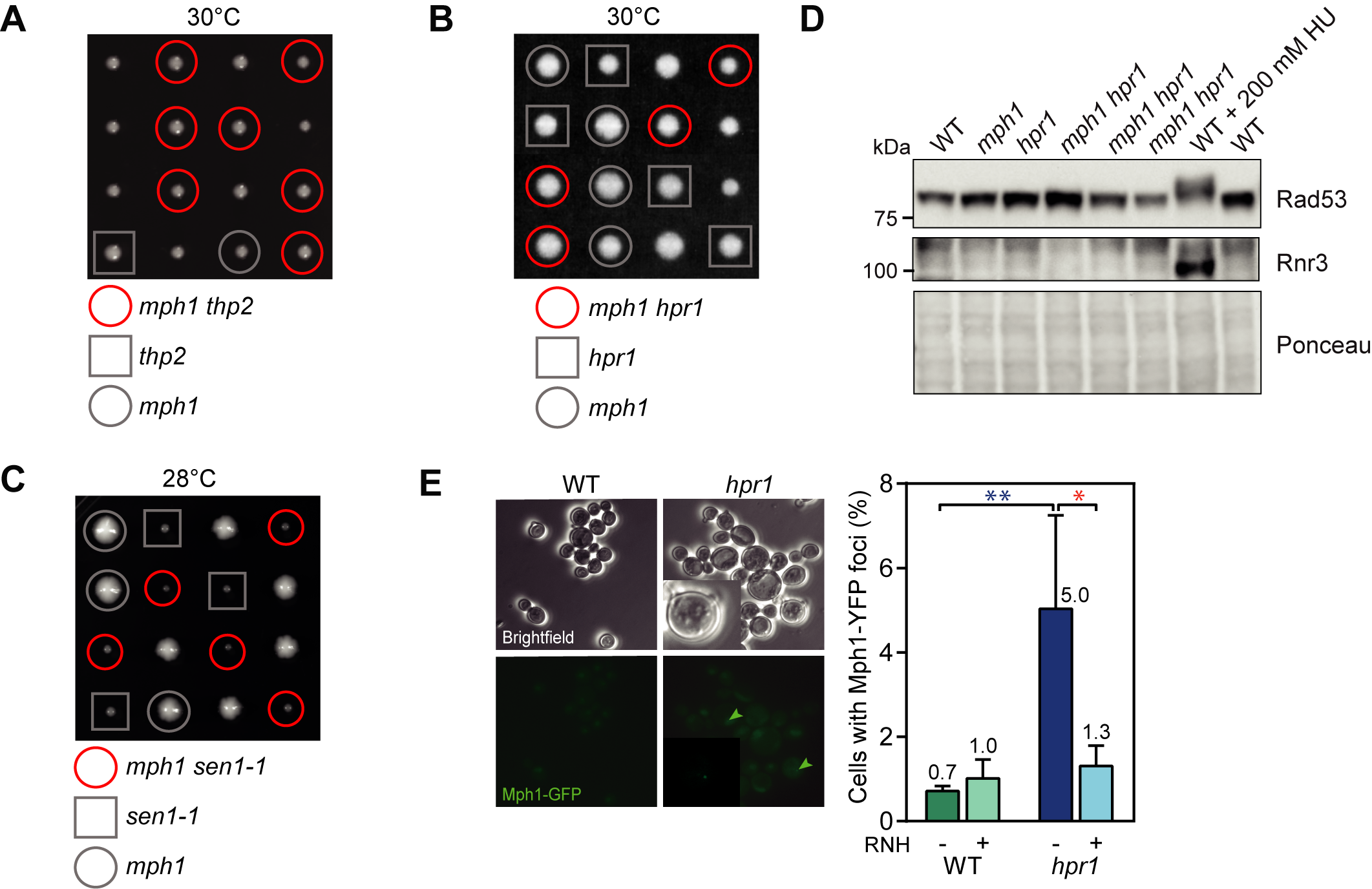

Supplement: S2 Fig — A. The heterozygous diploid THP2 MPH1/thp2 mph1 strain (YSLG649, left) was sporulated, micromanipulated and pictures were taken after two days incubation at 30°C. B. Tetrad analysis from crosses between mph1 (WMPH1-2B) and hpr1 (HPBAR-R1). Pictures were taken 4 days after micromanipulation at 30°C. C. Tetrad analysis of an mph1 sen1-1 mutant cross (yMG187). Pictures were taken after 3 days incubation at 28°C. D. anti-Rad53 and -Rnr3 Western Blot in three different hpr1 mph1 double mutants along with the single mutant controls. Treatment with HU served as positive controls for Rad53 phosphorylation and Rnr3 induction. Ponceau staining was used as loading control. E. Mph1-YFP foci accumulate in hpr1 cells as determined by microscopy in WT (MLL66-11A) and hpr1 (YBG722) cells following RNH1 overexpression (pCM189::RNH1). Left panel: representative image with arrowheads pointing to Mph1 foci; right panel: quantification. (*) P < 0.05; (**) P<0.01 (Student’s t-test). (TIF) [file pgen.1007136.s002.tif]

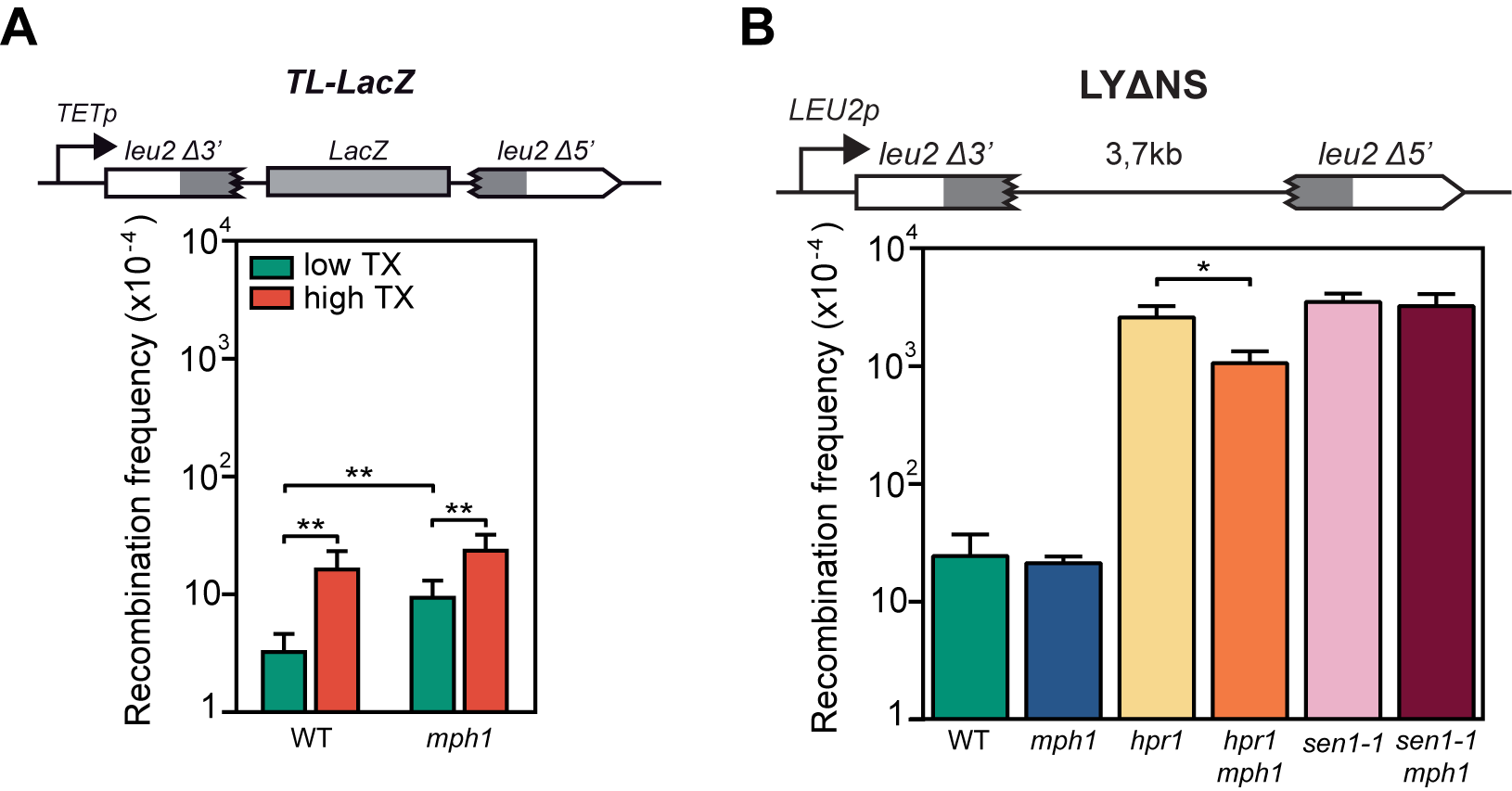

Supplement: S3 Fig — A. Recombination analysis of WT (W303-1A) and mph1 (WMPH1-2B) strains carrying the TL-lacZ plasmid system, whose transcription is regulated by the tet promoter, in the presence (low transcription) or absence (high transcription) of 5 mg/mL doxycycline. B. Recombination analysis of WT (W303.1A), mph1 (WMPH1.2B), hpr1 (HPBAR-R1), hpr1 mph1 (WMPHP.5A), sen1-1 (SEN1-R), sen1-1mph1 (WMPSEN.1C) strains carrying the LYΔNS plasmid. Leu+ recombinants resulting from recombination in TL-lacZ and LYΔNS systems were selected on SC-Leu plates. Mean and SD for at least three fluctuation tests consisting in the median value of six independent colonies each are shown. (*), P < 0.05, (**) P<0.01 (Student’s t-test). A scheme of the recombination system is shown on top. (TIF) [file pgen.1007136.s003.tif]

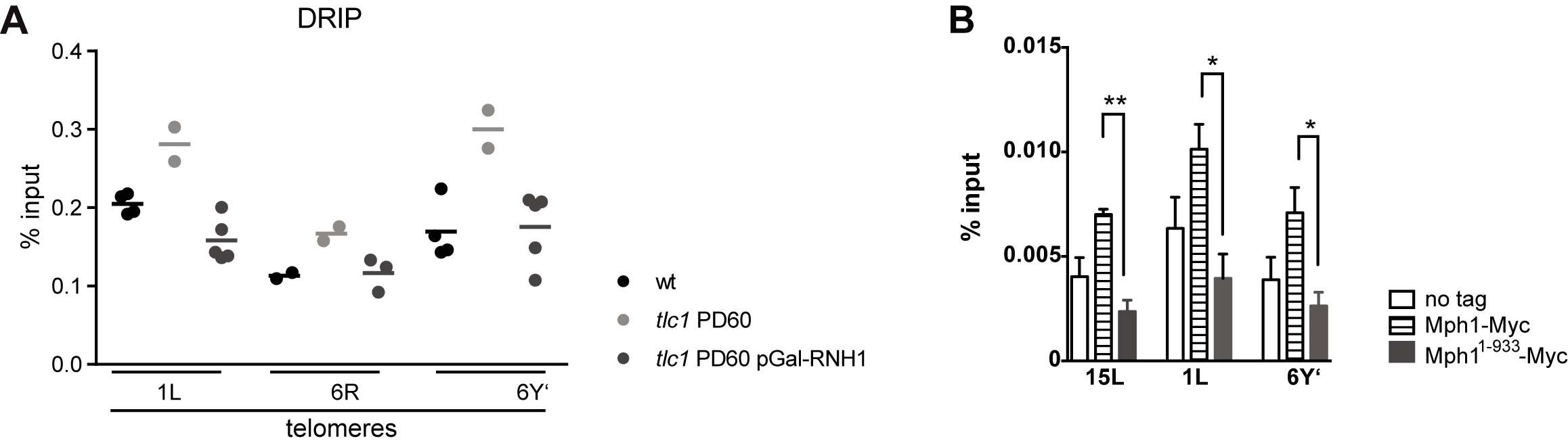

Supplement: S4 Fig — A. DRIP was performed with the S9.6 antibody in order to determine the RNA-DNA hybrid levels in senescing cells (PD60 tlc1) in the presence or absence of Rnh1. RNH1 was expressed endogenously under a galactose-inducible promoter (haploids derived from yMG103). Three different telomeres were analysed (1L, 6R and 6Y’). Individual qPCR data points are represented along with the mean (bar). B. Non-tagged wild type, Mph1-13myc (ySLG295) and Mph11-933-13myc (ySLG527) were grown to exponential phase in liquid YPD media at 30°C. Cells were cross-linked and ChIP was performed using anti-myc monoclonal antibodies. ChIP signals were quantified by qPCR specific to 15L, 1L and 6Y’ telomeres. Error bars represent 95% confidence intervals where n = 3 biological isolates were used for each indicated genotype. (p-values were calculated by a student’s test; * p<0.05, ** p<0.005). C. yAL331 was sporulated and the indicated meiotic products with the corresponding genotypes were spotted at the indicated temperatures on YPD and imaged following 48 h incubation. (TIF) [file pgen.1007136.s004.tif]

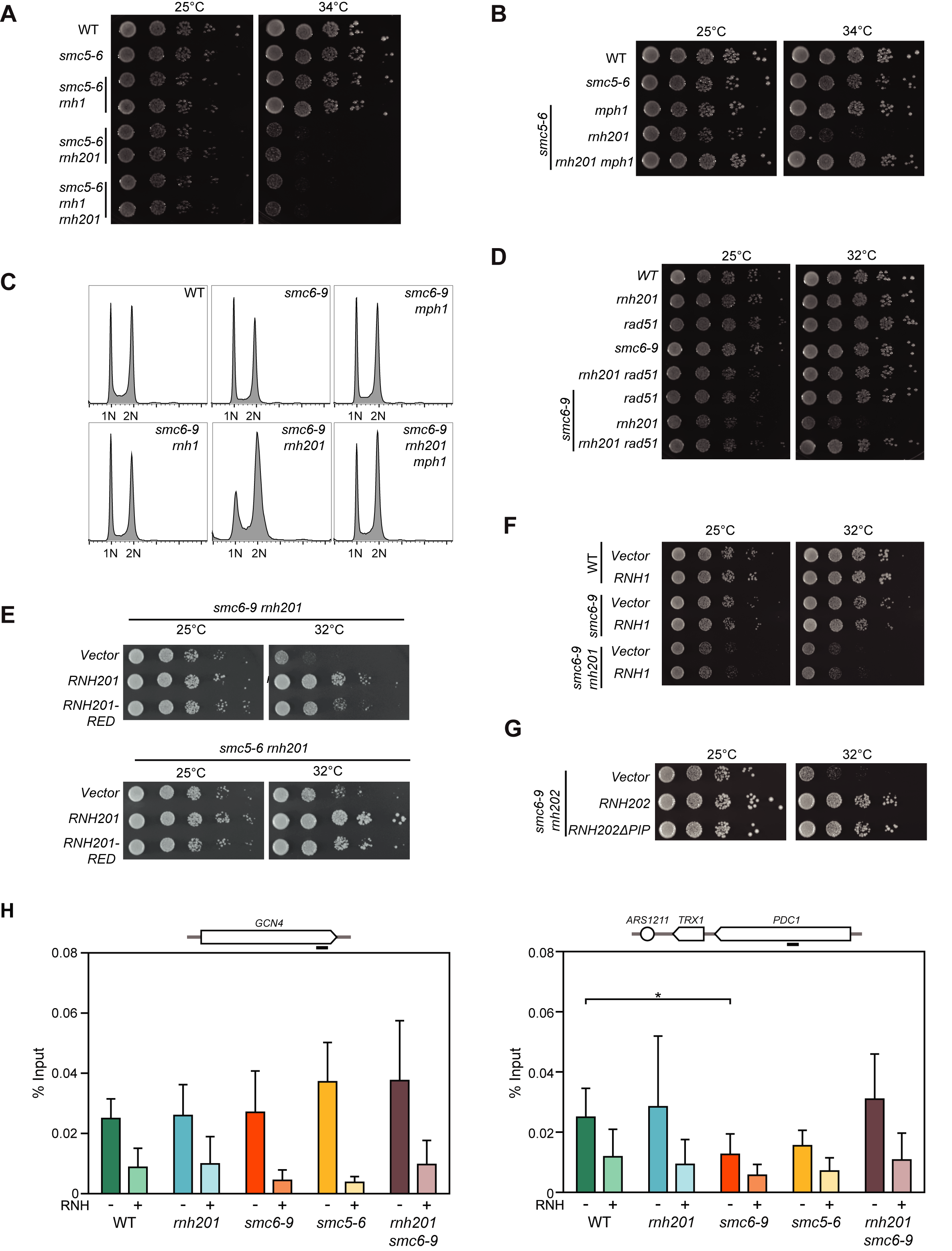

Supplement: S5 Fig — A. ySLG418 heterozygous diploids were microdissected and haploid offsprings were grown overnight in liquid YPD at 25°C and spotted as 10-fold serial dilutions onto YPD-agar at the indicated temperatures. Images were obtained after 2 days of incubation. B. The heterozygous diploid ySLG418 was microdissected to gain haploid offsprings. 10-fold serial dilutions were spotted onto YPD-agar at the indicated temperatures. C. DNA staining with Sytox Green coupled to flow cytometry was used to analyze the proportion of cells with a 1N and 2N DNA content. D. Yeast strains with the indicated genotypes were spotted as 10-fold serial dilutions onto YPD agar and incubated at the indicated temperatures following overnight growth in liquid YPD at 25°C. Images were acquired after 2 days of incubation. Haploids were derived from the heterozygous diploid strain yBL1021. E. smc6-9 rnh201 (upper panel) and smc5-6 rnh201 (lower panel) cells were transformed with an empty vector, or with plasmids containing either the RNH201 wild type or the RNH201-RED allele. Expression of both alleles alleviated the growth defects at 32°C. Cells were grown at the indicated temperatures for two days. F. A plasmid expressing RNH1 (pBB39) or an empty vector (pBL189) control were introduced into wild type, smc6-9 and smc6-9 rnh201 cells. The strains were grown at the indicated temperature for two days. G. RNH202 wild type (pBL506) and RNH202 lacking the PIP-box (pBL507) domain (RNH202 ΔPIP) were expressed from a plasmid in smc6-9 rnh202 cells. Cells were grown for 72 hours at the indicated temperatures. smc6-9 rnh202 cells carrying the empty vector (pBL505) served as control. H. DRIP with S9.6 antibody in WT (yBL7), rnh201 (yBL435), smc6-9 (ySLG88), smc5-6 (ySLG90) and rnh201 smc6-9 (ySLG397) strains in asynchronous cultures treated (+) or not (-) in vitro with RNase H in the GCN4 and PDC1 genes (left and right panels respectively). Data represent mean and SD of five independent experiments. * P < 0.05; (tw [file pgen.1007136.s005.tif]
