# Supplementary material for: The Smc5/6 complex regulates the yeast Mph1 helicase at RNA-DNA hybrid-mediated DNA damage
Source: PLoS Genet. 2017 Dec 27;13(12):e1007136. doi: 10.1371/journal.pgen.1007136 (PMC5760084; doi:10.1371/journal.pgen.1007136)
Supplement: S1 Table — This table lists the primers used in this study. (DOCX) [file pgen.1007136.s006.docx]

**S1 Table – Oligonucleotide primers used**

| oBL295 | 1L fwd | CGGTGGGTGAGTGGTAGTAAGTAGA |
| --- | --- | --- |
| oBL296 | 1L rev | ACCCTGTCCCATTCAACCATAC |
| oBL258 | 6R fwd | GTGTGTAGTGATCCGAACTCA |
| oBL259 | 6R rev | GCATATTGATATGGCGTACGCACACGT |
| oLK57 | 15L fwd | GGGTAACGAGTGGGGAGGTAA |
| oLK58 | 15L rev | CAACACTACCCTAATCTAACCCTGT |
| oLK49 | 6Y’ fwd | GGCTTGGAGGAGACGTACATG |
| oLK50 | 6Y’ rev | CTCGCTGTCACTCCTTACCCG |
| oBL292 | actin fwd | CCCAGGTATTGCCGAAAGAATGC |
| oBL293 | actin rev | TTTGTTGGAAGGTAGTCAAAGAAGCC |
| oAM47 | 18S rDNA fwd | TCCAATTGTTCCTCGTTAAG |
| oAM48 | 18S rDNA rev | ATTCAGGGAGGTAGTGACAA |
|  | Mph1(1-933)-YFP-fwd | ACGTTATTTAACGACGAGAGTAATGATAATAAGAAGAGGGCTGCAGCTGCAATGAGTAAAGGAG |
|  | Mph1-YFP-fwd | GATTACACATGGCATGGATGAACTATACAAATAGGAGACTCTTATACGT |
|  | Mph1(1-933)-YFP-rev | GTTCTTCTCCTTTACTCATTGCAGCTGCAGCCCTCTTCTTATTATCATTAC |
|  | Mph1-rev2 | CAGTGCCGTATCCTTAATGA |
|  | Mph1-down | GATGACACTATGTGACAGC |
| MX6-HPR1 up | MX6 -*HPR1* up | TAACAATTCAAGAGGCATTAAAACTTGGGCAAAGGAGTAATAATGGATCCCCGGGTTAATTAAGG |
| MX6-HPR1 down | MX6 -HPR1 down | ATGAATTTCTTATCAGTTTAAAATTTCTATTAAGAGGATAATTTATAGTGGATCTGATATCATCG |
|  | *MPH1*-MX6nt-Fw | CATTCCGGTTCTGTTTTATTTTAGTGTCCTTTTTTCTCTCTGATGCGGATCCCCGGGTTAATTAA |
|  | *MPH1*-MX6nt-Rv | AGCGTTATTTTTGTATAGACGCCGACGTATAAGAGTCTCCTATCATAGTGGATCTGATATCATCG |
