# Supplementary material for: The Smc5/6 complex regulates the yeast Mph1 helicase at RNA-DNA hybrid-mediated DNA damage
Source: PLoS Genet. 2017 Dec 27;13(12):e1007136. doi: 10.1371/journal.pgen.1007136 (PMC5760084; doi:10.1371/journal.pgen.1007136)
Supplement: S2 Table — This table lists the yeast strains employed in this study. (DOCX) [file pgen.1007136.s007.docx]

**S2 Table – yeast strains used**

| **BY4741** |  |  |
| --- | --- | --- |
| yAL95 | *MAT*a/α ; *his3Δ1*/ *his3Δ1*;*leu2Δ0* /*leu2Δ0*; *ura3Δ0*/*ura3Δ0*; *MET15*/ *met15Δ0*; *LYS2*/*lys2Δ0*; *TLC1*/*tlc1*::*NAT* | this study |
| yAL404 | *MAT*a*/alpha; his3Δ1/his3Δ1; leu2Δ0/leu2Δ0; ura3Δ0/ura3Δ0; met15Δ0/met15Δ0; RNH201/rnh201::HYG  rnh1::KAN/NAT-S-RNH1-TAP-HIS3 MPH1/mph1::HIS3* | this study |
| yAL296 | *MAT*a; *his3Δ1; leu2Δ0; ura3Δ0; met15Δ0; NAT-S-RNH1-TAP-HIS3* | this study |
| yBL7 | *MAT*a; his3Δ1; leu2Δ0; ura3Δ0; met15Δ0 | *Euroscarf* |
| yBL435 | *MAT*a *rnh201::KAN* | this study |
| yBL1047 | *MAT*α; *RAD52-mCherry-NAT; smc6-9-3HA-HIS3::KAN* | this study |
| yBL1048 | *MAT*a; *RAD52-mCherry-NAT; smc6-9-3HA-HIS3; rnh201::HYG* | this study |
| yBL1049 | *MAT*a; *RAD52-mCherry-NAT; mph1::KAN, smc6-9-3HA-HIS3; rnh201::HYG* | this study |
| yBL1050 | *MAT*a; *RAD52-mCherry-NAT; mph1::KAN; smc6-9-3HA-HIS3* | this study |
| yBL1051 | *MAT*a; *RAD52-mCherry-NAT; mph1::KAN* | this study |
| yBL1052 | *MAT*a; *RAD52-mCherry-NAT* | this study |
| yBL1053 | *MAT*α; *RAD52-mCherry-NAT, rnh201::HYG* | this study |
| yBL1060 | *MAT*a; *pol2 M644G-NAT; rnh201::HYG* |  |
| yBL1021 | *MAT*a/α; *RNH201/rnh201::HYG; RAD51/rad51::NAT; SMC6/smc6-9-3HA-HIS3* | this study |
| yBL1022 | *MAT*a/α; *RNH201/rnh201::HYG; RAD52/rad52::NAT; SMC6/smc6-9-3HA-HIS3* | this study |
| yBL 371 | *MAT*a; *rad53-11-URA3* | Kindly provided by Michael Chang (ERIBA) |
| ySLG2 | *MAT*a; *mph1::KAN* | this study |
| ySLG88 | *MAT*a; *SMC6::smc6-9-3HA-HIS3* | this study |
| ySLG90 | *MAT*a; *SMC5::smc5-6-3HA-HIS3* | this study |
| ySLG115 | *MAT*a/α; *SMC6/smc6-9-3HA-HIS3; EST2/est2::KAN; MPH1/mph1::NAT* | this study |
| ySLG251 | *MAT*a; *his3Δ1; leu2Δ0; ura3Δ0; met15Δ0; CDC13-TAP-HIS3MX6* | Dharmacon, GE healthcare |
| ySLG295 | *MATa*; *Mph1-13myc::HIS3* | this study |
| ySLG397 | *MAT*a; *rnh201::HYG; smc6-9::HIS3* | this study |
| ySLG418 | *MAT*a/α; *SMC5/smc5-6-HA-HIS3; RNH1/rnh1::KAN; RNH201/rnh201::HYG; MPH1/mph1::NAT* | this study |
| ySLG419 | *MAT*a/α; *SMC6/smc6-9-HA-HIS3; RNH1/rnh1::KAN; RNH201/rnh201::HYG; MPH1/mph1::NAT* | this study |
| ySLG428 | *MAT*a; *rnh1::KAN; rnh201::HYG* | this study |
| ySLG527 | *MAT*a; *Mph1^1-933^-13myc::HIS3* | this study |
| ySLG605 | *MAT*a/α; *RNH201/rnh201::HYG; RNH1/rnh1::KAN; MPH1/mph1::HIS* | this study |
| ySLG611 | *MAT*a; *rnh1::KAN; rnh201::HYG; mph1::HIS3* | this study |
| ySLG636 | *his3Δ1; leu2Δ0; ura3Δ0; met15Δ0; MPH1-GFP-HIS3* | this study |
| ySLG639 | *his3Δ1; leu2Δ0; ura3Δ0; met15Δ0 rnh1::KAN; MPH1-GFP-HIS* | this study |
| ySLG640 | *MAT*a; *MPH1-GFP-HIS3; rnh201::HYG* | this study |
| ySLG642 | *MAT*α; *MPH1-GFP-HIS3; rnh1::KAN; rnh201::HYG* | this study |
| ySLG649 | *MAT*a/α; *THP2/thp2::KAN; MPH1/mph1::HIS* | this study |
| ySLG661 | *MAT*a/α; *THP2/thp2::KAN; SMC6/smc6-9-3HA-HIS; MPH1/mph1::NAT* | this study |
| YDR138W | BY4741 *hpr1∆::KAN* | Euroscarf |
| YIR002C | BY4741 *mph1∆::KAN* | Euroscarf |
| YHR031C | BY4741 *rrm3∆::KAN* | Euroscarf |
| Y10775 | BY4741 *sgs1Δ::KAN* | Euroscarf |
| Y01331 | BY4741 *srs2Δ::KAN* | Euroscarf |
| yMG103 | *MAT*a/α; *his3Δ1/ his3Δ1;leu2Δ0 /leu2Δ0; ura3Δ0/ura3Δ0; MET15/ met15Δ0; RNH1/rnh1::pGAL1-3HA-RNH1-NAT* | this study |
| yMG156 | *MAT*a; *his3Δ1; leu2Δ0; ura3Δ0; met15Δ0*; pBL97 | this study |
| yMG157 | *MAT*a; *his3Δ1; leu2Δ0; ura3Δ0; met15Δ0*; pBL401 | this study |
| yMG158 | *MAT*a; *his3Δ1; leu2Δ0; ura3Δ0; met15Δ0*; pBL399 | this study |
| yMG159 | *MAT*a; *his3Δ1; leu2Δ0; ura3Δ0; met15Δ0; mph1::HIS3; rnh1::KAN; rnh201::HYG*; pBL97 | this study |
| yMG160 | *MAT*a; *his3Δ1; leu2Δ0; ura3Δ0; met15Δ0; mph1::HIS3; rnh1::KAN; rnh201::HYG*; pBL401 | this study |
| yMG161 | *MAT*a; *his3Δ1; leu2Δ0; ura3Δ0; met15Δ0; mph1::HIS3; rnh1::KAN; rnh201::HYG*; pBL399 | this study |
| yMG164 | *MAT*a; *his3Δ1; leu2Δ0; ura3Δ0; met15Δ0*; pBL301 | this study |
| yMG165 | *MAT*a; *his3Δ1; leu2Δ0; ura3Δ0; met15Δ0*; pBL472 | this study |
| yMG166 | *MAT*a; *his3Δ1; leu2Δ0; ura3Δ0; met15Δ0*; pBL473 | this study |
| yMG167 | *MAT*a; *his3Δ1; leu2Δ0; ura3Δ0; met15Δ0*; pBL474 | this study |
| yMG168 | *MAT*a; *his3Δ1; leu2Δ0; ura3Δ0; met15Δ0; mph1::HIS3; rnh1::KAN; rnh201::HYG*; pBL301 | this study |
| yMG169 | *MAT*a; *his3Δ1; leu2Δ0; ura3Δ0; met15Δ0; mph1::HIS3; rnh1::KAN; rnh201::HYG*; pBL472 | this study |
| yMG170 | *MAT*a; *his3Δ1; leu2Δ0; ura3Δ0; met15Δ0; mph1::HIS3; rnh1::KAN; rnh201::HYG*; pBL473 | this study |
| yMG171 | *MAT*a; *his3Δ1; leu2Δ0; ura3Δ0; met15Δ0; mph1::HIS3; rnh1::KAN; rnh201::HYG*; pBL474 | this study |
| yMG187 | *MAT*a/α; *his3Δ1/ his3Δ1*; *leu2Δ0 /leu2Δ0; ura3Δ0/ura3Δ0; met15Δ0/met15Δ0;* *MPH1/mph1::NAT*; *SEN1/sen1-1-KAN* | this study |
| yMG195 | *MAT*a; *his3Δ1; leu2Δ0; ura3Δ0; met15Δ0; smc6-9-3HA-HIS3; rnh201::HYG; mph1::KAN;* pBL301 | this study |
| yMG196 | *MAT*a; *his3Δ1; leu2Δ0; ura3Δ0; met15Δ0; smc6-9-3HA-HIS3; rnh201::HYG; mph1::KAN*; pBL472 | this study |
| yMG197 | *MAT*a; *his3Δ1; leu2Δ0; ura3Δ0; met15Δ0; smc6-9-3HA-HIS3; rnh201::HYG; mph1::KAN;* pBL473 | this study |
| yMG198 | *MAT*a; *his3Δ1; leu2Δ0; ura3Δ0; met15Δ0; smc6-9-3HA-HIS3; rnh201::HYG; mph1::KAN;* pBL474 | this study |
| yLP61 | *MAT*a; *his3Δ1; leu2Δ0; ura3Δ0; met15Δ0; Rad52-TAP-HIS3MX6* | Dharmacon, GE healthcare |
| yAL331 | *MAT*a/α; *his3Δ1/his3Δ1; leu2Δ0/leu2Δ0; ura3Δ0/ura3Δ0; met15Δ0/met15Δ0; SMC6/smc6-9::HIS RNH201/rnh201::HYG MTE1/mte1::KAN* | this study |
| **W303 strains**  W303-1AR5 | *MAT*a; *ade2-1; leu2-3,112; trp1-1; can1-100; ura3-1; his3-11,15; RAD5* | [1] |
| WMPH1-2B | *MAT*a; *mph1∆::KAN* | this study |
| HPBAR-R1 | *MAT*a; *bar1::HYG; hpr1∆3::HIS3* | kindly provided by Marta San Martin Alonso (MSMA) |
| WMPHP-5A | *MAT*α; *mph1∆::KAN; hrp1∆HIS3* | this study |
| SEN1-R | *MAT*a; *bar1::HYG; sen1-1* | MSMA |
| WMPSEN-1C | *MAT*α; *mph1∆::KAN; sen1-1* | this study |
| T597-1 | *MAT*α; *mph1-Q603D-YFP::HIS5* | [2] |
| T617 | *MAT*α; *mph1-E210Q-YFP::HIS5* | [2] |
| RNH-R | *MAT*a; *bar1::HYG; rnh1::KAN; rnh201::KAN* | MSMA |
| ML66-11A | *MAT*a; *can1-100; ura3-1; his3-11,15; leu2-3, 112; trp1-1;*  *MPH1-YFP* | [3] |
| YBG722 | *MAT*a; *can1-100; ura3-1; his3-11,15; leu2-3, 112; trp1-1;*  *MPH1-YFP;* *hpr1∆::KAN* | this study |
| Ybpr1.1 | W303.1B *bar1Δ; rnh1::NAT* | M. García-Rubio |
| Ybpr1m1.1 | W303.1B *bar1Δ; rnh1::NAT; mph1::HYG* | This study |
| rnh1rnh201.8D | *MAT*a; *ADE2; leu2-3,112; trp1-1; can1-100; ura3-1; his3-11,15; rnh1::NAT; rnh201::KAN* | M. San Martin-Alonso |
| **W1588-4C**  SS281-8B | *MAT*a; *MPH1-YFP; smc6-9::NAT; cdc13-1* | this study |
| SS283-23D | *MAT*a; *ura3-1; MPH1-YFP; CDC13-CFP; RAD52-RFP; est2::KAN* | this study |

**References:**

1. Moriel-Carretero M, Aguilera A. A postincision-deficient TFIIH causes replication fork breakage and uncovers alternative Rad51- or Pol32-mediated restart mechanisms. Molecular cell. 2010;37(5):690-701. doi: 10.1016/j.molcel.2010.02.008. PubMed PMID: 20227372.

2. Chen YH, Choi K, Szakal B, Arenz J, Duan X, Ye H, et al. Interplay between the Smc5/6 complex and the Mph1 helicase in recombinational repair. Proceedings of the National Academy of Sciences of the United States of America. 2009;106(50):21252-7. Epub 2009/12/10. doi: 10.1073/pnas.0908258106. PubMed PMID: 19995966; PubMed Central PMCID: PMC2795505.

3. Silva S, Altmannova V, Luke-Glaser S, Henriksen P, Gallina I, Yang X, et al. Mte1 interacts with Mph1 and promotes crossover recombination and telomere maintenance. Genes & development. 2016;30(6):700-17. Epub 2016/03/12. doi: 10.1101/gad.276204.115. PubMed PMID: 26966248; PubMed Central PMCID: PMC4803055.
