# Supplementary material for: The Smc5/6 complex regulates the yeast Mph1 helicase at RNA-DNA hybrid-mediated DNA damage
Source: PLoS Genet. 2017 Dec 27;13(12):e1007136. doi: 10.1371/journal.pgen.1007136 (PMC5760084; doi:10.1371/journal.pgen.1007136)
Supplement: S3 Table — Plasmids used during this analysis are summarized in S3 Table. (DOCX) [file pgen.1007136.s008.docx]

**S3 Table – Plasmid list**

| **Code** | **Name** | **Genotype** | | **Source** |  |
| --- | --- | --- | --- | --- | --- |
| pAP81 | *EST2* | *EST2, URA3* | [1] | | |
| pBB39 | *RNH1, URA3* | pRS426, *pGPD-RNH1-HA, URA3* | [2] | | |
| pBL97 | Empty vector control for pBL399 and 401 | pRS316, *URA3* | Gift from Matthias Peter | | |
| pBL189 | Empty vector control for pBB39 | pRS426 pGPD, 2µ, *URA3* | [2] | | |
| pBL190 | Empty vector control for pBL192 | pRS423, *pGPD, HIS3* | Gift from Matthias Peter | | |
| pBL192 | *RNH1* | pRS423, *pGPD-RNH1, HIS3* | [2] | | |
| pBL301 | empty vector control for pBL472-474 | pRS41N; NAT | Kind gift from  M. Knop | | |
| pBL399 | *RNH201*  *P45D,Y219A* | pRS316, *RNH201 P45D, Y219A, URA3* | [3] | | |
| pBL401 | *RNH201* | pRS316, *RNH201, URA3* | [3] | | |
| pBL472 | *MPH1-1xHA* | pRS41N; MPH1-1xHA; NAT | this study | | |
| pBL473 | *mph1-E210Q-1xHA* | pRS41N; mph1-E210Q-1xHA; NAT | this study | | |
| pBL474 | *mph1-Q603-1xHA* | pRS41N; mph1-Q603D-1xHA; NAT | this study | | |
| pBL505 | empty vector control for pBL506 - 507 | CEN, *LEU2*  *(YCplac111)* | Gift from Robert J. Crouch | | |
| pBL506 | *RNH202* | CEN, *RNH202*, *LEU2* | Gift from Robert J. Crouch | | |
| pBL507 | *RNH202ΔPIP* | CEN, *RNH202ΔPIP*, *LEU2* | Gift from Robert J. Crouch | | |
| pWJ1314 | Vector for *RAD52-YFP* counting | CEN/ARS, *LEU2*, *RAD52-YFP* | [4] | | |
| pCM189 | Empty vector control for *RNH1* overexpression | CEN/ARS, *URA3, ptet* | [5] | | |
| pCM189::RNH1 | Vector for *RNH1* overexpression | CEN/ARS, *URA3, ptet::RNH1* | [6] | | |
| TL-LacZ | YCp pCM189 for the TL-LacZ recombination assay | CEN/ARS, *URA3, ptet:leu2∆3‘::lacZ:.leu2∆5‘* | [7] | | |
| LY∆NS | Plasmid for the LY∆NS recombination assay | pRS316, *TRP, LY∆NS* recombination system | [8] | | |
| pRS315 | Vector for control | CEN/ARS, *LEU2* | [9] | | |
| pRS315-GAL:RNH1 | Vector for *RNH1* expression from *pGAL* | CEN/ARS, *LEU2, pGAL::RNH1* | [10] | | |
| pFA6-KANMX6 |  | Plasmid carrying the *KanMX4* selector module | [11] | | |

**References:**

1. Khadaroo B, Teixeira MT, Luciano P, Eckert-Boulet N, Germann SM, Simon MN, et al. The DNA damage response at eroded telomeres and tethering to the nuclear pore complex. Nature cell biology. 2009;11(8):980-7. Epub 2009/07/15. doi: ncb1910 [pii]

10.1038/ncb1910. PubMed PMID: 19597487.

2. Balk B, Maicher A, Dees M, Klermund J, Luke-Glaser S, Bender K, et al. Telomeric RNA-DNA hybrids affect telomere-length dynamics and senescence. Nature structural & molecular biology. 2013;20(10):1199-205. Epub 2013/09/10. doi: 10.1038/nsmb.2662. PubMed PMID: 24013207.

3. Graf M, Bonetti D, Lockhart A, Serhal K, Kellner V, Maicher A, et al. Telomere Length Determines TERRA and R-Loop Regulation through the Cell Cycle. Cell. 2017;170(1):72-85 e14. Epub 2017/07/01. doi: 10.1016/j.cell.2017.06.006. PubMed PMID: 28666126.

4. Lisby M, Rothstein R, Mortensen UH. Rad52 forms DNA repair and recombination centers during S phase. Proceedings of the National Academy of Sciences of the United States of America. 2001;98(15):8276-82. doi: 10.1073/pnas.121006298. PubMed PMID: 11459964; PubMed Central PMCID: PMCPMC37432.

5. Gari E, Piedrafita L, Aldea M, Herrero E. A set of vectors with a tetracycline-regulatable promoter system for modulated gene expression in Saccharomyces cerevisiae. Yeast. 1997;13(9):837-48. doi: 10.1002/(SICI)1097-0061(199707)13:9<837::AID-YEA145>3.0.CO;2-T. PubMed PMID: 9234672.

6. Castellano-Pozo M, Santos-Pereira JM, Rondon AG, Barroso S, Andujar E, Perez-Alegre M, et al. R loops are linked to histone h3 s10 phosphorylation and chromatin condensation. Molecular cell. 2013;52(4):583-90. Epub 2013/11/12. doi: 10.1016/j.molcel.2013.10.006. PubMed PMID: 24211264.

7. Santos-Pereira JM, Herrero AB, Garcia-Rubio ML, Marin A, Moreno S, Aguilera A. The Npl3 hnRNP prevents R-loop-mediated transcription-replication conflicts and genome instability. Genes & development. 2013;27(22):2445-58. Epub 2013/11/19. doi: 10.1101/gad.229880.113. PubMed PMID: 24240235; PubMed Central PMCID: PMC3841734.

8. Prado F, Piruat JI, Aguilera A. Recombination between DNA repeats in yeast hpr1delta cells is linked to transcription elongation. The EMBO journal. 1997;16(10):2826-35. doi: 10.1093/emboj/16.10.2826. PubMed PMID: 9184227; PubMed Central PMCID: PMCPMC1169891.

9. Sikorski RS, Hieter P. A system of shuttle vectors and yeast host strains designed for efficient manipulation of DNA in Saccharomyces cerevisiae. Genetics. 1989;122(1):19-27. PubMed PMID: 2659436; PubMed Central PMCID: PMCPMC1203683.

10. Gomez-Gonzalez B, Garcia-Rubio M, Bermejo R, Gaillard H, Shirahige K, Marin A, et al. Genome-wide function of THO/TREX in active genes prevents R-loop-dependent replication obstacles. The EMBO journal. 2011;30(15):3106-19. Epub 2011/06/28. doi: emboj2011206 [pii]

10.1038/emboj.2011.206. PubMed PMID: 21701562; PubMed Central PMCID: PMC3160181.

11. Bahler J, Wu JQ, Longtine MS, Shah NG, McKenzie A, 3rd, Steever AB, et al. Heterologous modules for efficient and versatile PCR-based gene targeting in Schizosaccharomyces pombe. Yeast. 1998;14(10):943-51. doi: 10.1002/(SICI)1097-0061(199807)14:10<943::AID-YEA292>3.0.CO;2-Y. PubMed PMID: 9717240.
